# The Vancomycin Resistance-Associated Regulatory System VraSR Modulates Biofilm Formation of Staphylococcus epidermidis in an *ica*-Dependent Manner

**DOI:** 10.1128/mSphere.00641-21

**Published:** 2021-09-22

**Authors:** Youcong Wu, Yuanyuan Meng, Lian Qian, Baixing Ding, Haiyan Han, Hongling Chen, Li Bai, Di Qu, Yang Wu

**Affiliations:** a Department of Medical Microbiology and Immunology, School of Basic Medical Sciences, Dali University, Dali, Yunnan Province, China; b Key Laboratory of Medical Molecular Virology (MOE/NHC/CAMS), School of Basic Medical Sciences, Shanghai Medical College, Fudan University, Shanghai, China; c Department of Laboratory Medicine, Second Affiliated Hospital of Zhengzhou University, Zhengzhou, Henan Province, China; University of Nebraska Medical Center

**Keywords:** two-component regulatory system, VraSR, biofilm formation, cell death, *Staphylococcus epidermidis*

## Abstract

The two-component system VraSR responds to the cell wall-active antibiotic stress in Staphylococcus epidermidis. To study its regulatory function in biofilm formation, a *vraSR* deletion mutant (Δ*vraSR*) was constructed using S. epidermidis strain 1457 (SE1457) as the parent strain. Compared to SE1457, the Δ*vraSR* mutant showed impaired biofilm formation both *in vitro* and *in vivo* with a higher ratio of dead cells within the biofilm. Consistently, the Δ*vraSR* mutant produced much less polysaccharide intercellular adhesin (PIA). The Δ*vraSR* mutant also showed increased susceptibility to the cell wall inhibitor and SDS, and its cell wall observed under a transmission electron microscope (TEM) appeared to be thinner and interrupted, which is in accordance with higher susceptibility to the stress. Complementation of *vraSR* in the Δ*vraSR* mutant restored the biofilm formation and the cell wall thickness to wild-type levels. Transcriptome sequencing (RNA-Seq) showed that the *vraSR* deletion affected the transcription levels of 73 genes, including genes involved in biofilm formation, bacterial programmed cell death (CidA-LrgAB system), glycolysis/gluconeogenesis, the pentose phosphate pathway (PPP), and the tricarboxylic acid (TCA) cycle, etc. The results of RNA-Seq were confirmed by quantitative real-time reverse transcription-PCR (qRT-PCR). In the Δ*vraSR* mutant, the expression of *icaA* and *lrgAB* was downregulated and the expression of *icaR* and *cidA* was upregulated, in comparison to that of SE1457. The transcriptional levels of antibiotic-resistant genes (*pbp2*, *serp1412*, *murAA*, etc.) had no significant changes. An electrophoretic mobility shift assay further revealed that phosphorylated VraR bound to the promoter regions of the *ica* operon, as well as its own promoter region. This study demonstrates that in S. epidermidis, VraSR is an autoregulator and directly regulates biofilm formation in an *ica*-dependent manner. Upon cell wall stress, it indirectly regulates cell death and drug resistance in association with alterations to multiple metabolism pathways.

**IMPORTANCE** S. epidermidis is a leading cause of hospital-acquired catheter-related infections, and its pathogenicity depends mostly on its ability to form biofilms on implants. The biofilm formation is a complex procedure that involves multiple regulating factors. Here, we show that a vancomycin resistance-associated two-component regulatory system, VraSR, plays an important role in modulating S. epidermidis biofilm formation and tolerance to stress. We demonstrate that S. epidermidis VraSR is an autoregulated system that selectively responds to stress targeting cell wall synthesis. Besides, phosphorylated VraR can bind to the promoter region of the *ica* operon and directly regulates polysaccharide intercellular adhesin production and biofilm formation in S. epidermidis. Furthermore, VraSR may indirectly modulate bacterial cell death and extracellular DNA (eDNA) release in biofilms through the CidA-LrgAB system. This work provides a new molecular insight into the mechanisms of VraSR-mediated modulation of the biofilm formation and cell death of S. epidermidis.

## INTRODUCTION

Staphylococcus epidermidis is an opportunistic pathogen that is a common resident on human skin and mucosal surfaces (1, 2). The threat of S. epidermidis is due in large part to the propensity to form adherent biofilms on indwelling medical devices, such as vascular catheters, artificial heart valves, and prosthetic joints, etc. (3, 4). The bacterial cells within the biofilm are protected against killing by antibiotics and the host immune system, leading to the increasing emergence of resistance to antimicrobial drugs and the establishment of persistent infections (5). The two-component system VraSR (vancomycin resistance-associated sensor-regulator system) positively modulates the regulation of the cell wall biosynthesis pathway in Staphylococcus aureus and plays a central role in maintaining the integrity of the cell wall peptidoglycan, antibiotic resistance against cell wall-active antibiotics, and expression of virulence factors (6, 7). However, the mechanisms by which staphylococcal VraSR regulates biofilm formation have not been investigated in great detail.

The VraSR, as a “sentinel” system in S. aureus, is capable of rapidly sensing cell wall damage and regulating the transcription of a series of genes related to peptidoglycan synthesis (8–11), such as *pbp2* (penicillin-binding protein), *sgtB* (monofunctional glycosyltransferase), and *murZ* (UDP-*N*-acetylglucosamine enolpyruvyl transferase), etc., and coordinates a response that enhances the resistance phenotype. Studies (12, 13) have shown that the *vraSR* knockout mutant strain of S. aureus has decreased resistance to methicillin, vancomycin, and daptomycin cell wall antimicrobials, exhibits a thinner cell wall under a scanning electron microscope (SEM), and is more susceptible to phagocytosis by polymorphonuclear leukocytes (PMNs) (6, 7). It is therefore possible that the inhibitor of VraSR could resensitize methicillin-resistant S. aureus (MRSA) to methicillin. Besides regulating drug susceptibility, S. aureus VraSR also modulates the expression of various virulence factors. Dai et al. (14) found that the expression of VraSR in vancomycin-intermediate S. aureus (VISA) and heterogeneous VISA (hVISA strains10827, Mu3, and Mu50) was upregulated; in contrast, the expression of virulence-related genes (*hla*, *hlb*, *coa*, *RNAIII*, *agrA*, *saeR*) is downregulated. However, the DNA-binding regulator VraR could not bind to any of the promoter regions of the virulence factor genes in VISA/hVISA, based on an electrophoretic mobility shift assay (EMSA), which indicated that VraSR indirectly regulated the transcription of virulence factor genes. Additionally, studies (12, 15–17) have found that YvqF/VraSR of S. aureus (MRSA) serves as an on-off switch in drug resistance and virulence factor phenotype. The deletion of *yvqF* located upstream of *vraSR* turned on the VraSR system and then increased drug resistance and decreased transcription of virulence-related genes (*agrA*, *rot*, *sarH1*, *spa*, α-hemolysin, etc.). However, the deletion of *vraS* turned off the VraSR system, and then the MRSA strain lost its drug-resistant phenotype and recovered the transcription level of these virulence genes.

In contrast to S. aureus, which is much more virulent and synthesizes many exotoxins and invasive enzymes, the pathogenicity of S. epidermidis is mainly due to its ability to colonize and form biofilm on biomaterials (3). Staphylococcal biofilm formation is typically considered a three-phase process to form highly ordered bacterial communities, consisting of attachment, accumulation, and maturation (18–20). The bacterial cells attached to polymer surfaces in the initial adhesion phase are influenced by many factors in S. epidermidis, such as AtlE, Embp, SdrG, and other staphylococcal surface-associated proteins. In the aggregation and maturation phases of S. epidermidis biofilm formation, the most important adhesive biofilm matrix is polysaccharide intercellular adhesin (PIA). PIA is synthesized by the *icaADBC* operon gene products; meanwhile, *icaA* is negatively regulated by the divergently transcribed *icaR* gene. Additionally, transcriptional regulation of the *icaADBC* operon has been extensively studied, and multiple factors function to modulate its expression, including SrrA, ArlR, SarA, RsbU, etc. (21–23); in addition, the tricarboxylic acid (TCA) cycle also partly participates in modulation of PIA production (24, 25).

Bioinformatics analysis further showed that although the VraS/VraR system in S. epidermidis strain RP62A shared about 92% identity with that in S. aureus strain Mu50 at the amino acid level, there are variations in the extracellular sensor domain of VraS proteins and in the CheY homologous receiver domain of VraR proteins in the two species, indicating that the VraSR system of S. epidermidis may have diverse functions (such as biofilm formation, environmental stress sensing, and drug resistance, etc.) with that of S. aureus. Much attention has been focused on the relevance of VraSR to drug resistance, cell wall synthesis, and virulence factors in S. aureus, while the mechanism by which staphylococcal VraSR regulates biofilm formation has not been investigated in great detail. Here, we have constructed the *vraSR* deletion mutant strain by allelic replacement in S. epidermidis 1457 and discovered new aspects of the role of VraSR in regulating biofilm formation and drug resistance in S. epidermidis.

## RESULTS

### S. epidermidis VraSR selectively responded to cell wall-active antibiotic stress.

**FIG 1:**
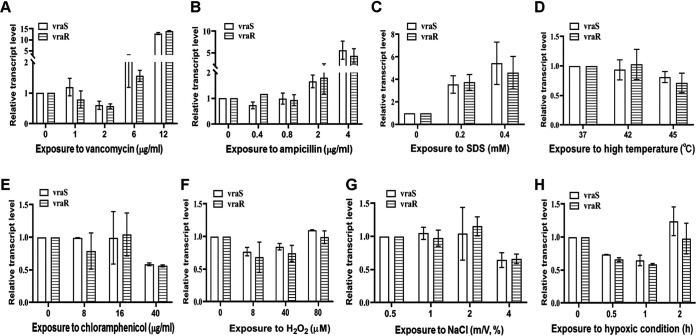
Transcriptional levels of *vraS* and *vraR* in SE1457 under diverse stresses. After culturing for 4 h, staphylococci were treated with different concentrations of vancomycin, ampicillin, chloramphenicol, H_2_O_2_, NaCl, or SDS for 30 min of incubation. Under microaerobic or heat stress, cultures were transferred into a 50-ml syringe (sealed entirely, with no bubbles inside) or shifted from 37°C to 42°C or 45°C, respectively. Bacterial cells were collected, and total RNA was extracted. The relative expression levels of *vraS* and *vraR* were analyzed by qRT-PCR in comparison to the transcription level of *gyrB* (housekeeping gene). Data are represented as means ± standard deviations (SD) of results from three independent experiment.

To assess whether *vraSR* expression responds to environmental stress, the transcription of *vraSR* in S. epidermidis strain 1457 (SE1457) treated with diverse stresses was analyzed by quantitative real-time reverse transcription-PCR (qRT-PCR). Both *vraS* and *vraR* expression were upregulated (2-fold and 13-fold increases, respectively) under the stress of cell wall-active agents (vancomycin, ampicillin, and the anionic surfactant SDS) but showed no obvious change under the pressure of H_2_O_2_, NaCl, hypoxia, heat, or chloramphenicol (Fig. 1), indicating that VraSR plays an important role in bacterial adaptation to environmental stress, especially cell wall stress.

### Deletion of *vraSR* increased susceptibility to antibiotics.

To identify the biological function of *vraSR* in S. epidermidis, a *vraSR* deletion mutant of the SE1457 strain was constructed by allelic replacement using the temperature-sensitive plasmid pKOR1 (see Fig. S1 in the supplemental material). The mutant was verified by PCR and reverse transcription (RT)-quantitative PCR (qPCR) and designated the Δ*vraSR* mutant. A *vraSR* gene complementation strain was then constructed using shuttle vector pRAB11 and named the Δ*vraSR*(pRAB11-*vraSR*) strain. The vector pRAB11 was introduced into the Δ*vraSR* and SE1457 strains, and the resulting Δ*vraSR*(pRAB11) and SE1457(pRAB11) strains, respectively, served as vector controls.

10.1128/mSphere.00641-21.1 FIG S1 Construction of *vraSR* deletion in SE1457 by allelic replacement. (A) The downstream (972-bp) and upstream (920-bp) flanking sequences of *vraSR* were amplified from SE1457 genomic DNA and cloned into the EcoRI site of pET28a, respectively, thus yielding recombinant plasmid pET28a-Δ*vraSR*. The fragment containing both the downstream and upstream regions was amplified with primers containing attB1 and attB2 sites at the 5′ end using pET28aΔ*vraSR* as a template and was inserted into pKOR1 using the BP Clonase enzyme, yielding the recombinant plasmid pKOR1Δ*vraSR*, which was then transformed into SE1457 for allelic replacement. (B) Confirmation of the *vraSR* deletion mutant by PCR using the primer pair vra-con-F/vra-con-R. Lane 1, genomic DNA of SE1457 parent strain was designated as a template; lanes 2 and 3, genomic DNA of the *vraSR* deletion mutants 1 and 2 as the templates; lane 4, DNA marker III. Due to a 1,644-bp deletion of *vraSR* genes, the PCR fragment amplified from the *vraSR* deletion mutant was smaller than that of SE1457. Download FIG S1, TIF file, 0.5 MB. Copyright © 2021 Wu et al. 2021 Wu et al. https://creativecommons.org/licenses/by/4.0/ This content is distributed under the terms of the Creative Commons Attribution 4.0 International license.

The impact of *vraSR* deletion on the susceptibility of S. epidermidis was determined by the serial broth dilution method and the disk diffusion method. The MICs of ampicillin and vancomycin for the Δ*vraSR* mutant were 0.1 to 0.2 μg/ml and 0.5 to 1 μg/ml, respectively, while they were 0.4 μg/ml and 4 μg/ml for parent strain SE1457. Gene complementation by plasmid expressing *vraSR* in the Δ*vraSR* mutant restored the MIC values to wild-type levels, whereas transformation of the vector alone had no obvious effect. The susceptibility levels of the SE1457, Δ*vraSR*, Δ*vraSR*(pRAB11-vraSR), and Δ*vraSR*(pRAB11) strains against kanamycin, chloramphenicol, erythromycin, and levofloxacin showed no significant differences (Table 1).

**TABLE 1:** Antimicrobial susceptibility of the S. epidermidis *vraSR* deletion mutant (broth dilution susceptibility test)

Strain	MIC (μg/ml)^*a*^					
	Am	Van	Km	Cm	Em	LVF
SE1457	0.4	4	1.6	5	0.4	0.5
Δ*vraSR* mutant	0.1–0.2	0.5-1	1.6	5	0.4	0.5
Δ*vraSR*(pRAB11-*vraSR*) mutant	0.4–0.8	4	1.6	5	0.4	0.5
Δ*vraSR*(pRAB11) mutant	0.1	0.5-1	1.6	5	0.4	0.5

a Am, ampicillin; Van, vancomycin; Km, kanamycin; Cm, chloramphenicol; Em, erythromycin; LVF, levofloxacin.

In accordance with the results detected by the serial broth dilution method, the Δ*vraSR* mutant displayed increased susceptibility to the antibiotics targeting cell wall biosynthetic pathways, such as vancomycin, ampicillin, cefuroxime, and cefotaxime. The Δ*vraSR*(pRAB11) strain was also more susceptible to the cell wall-active antibiotics than the SE1457(pRAB11) strain, and susceptibility was restored in the Δ*vraSR*(pRAB11-*vraSR*) complementation strain. However, the susceptibility of the *vraSR* deletion mutant to the other classes of antibiotics (amikacin, gentamicin, tetracycline, and levofloxacin) showed no significant difference from that of the parent strain (Table S1).

10.1128/mSphere.00641-21.5 TABLE S1 Antimicrobial susceptibility of the S. epidermidis *vraSR* deletion mutant (disk diffusion test). Van, vancomycin; Am, ampicillin; CXM, cefuroxime; CTX, cefotaxime; AK, amikacin; GN, gentamycin; TE, tetracycline; LVF, levofloxacin. Download Table S1, DOCX file, 0.02 MB. Copyright © 2021 Wu et al. 2021 Wu et al. https://creativecommons.org/licenses/by/4.0/ This content is distributed under the terms of the Creative Commons Attribution 4.0 International license.

**FIG 2:**
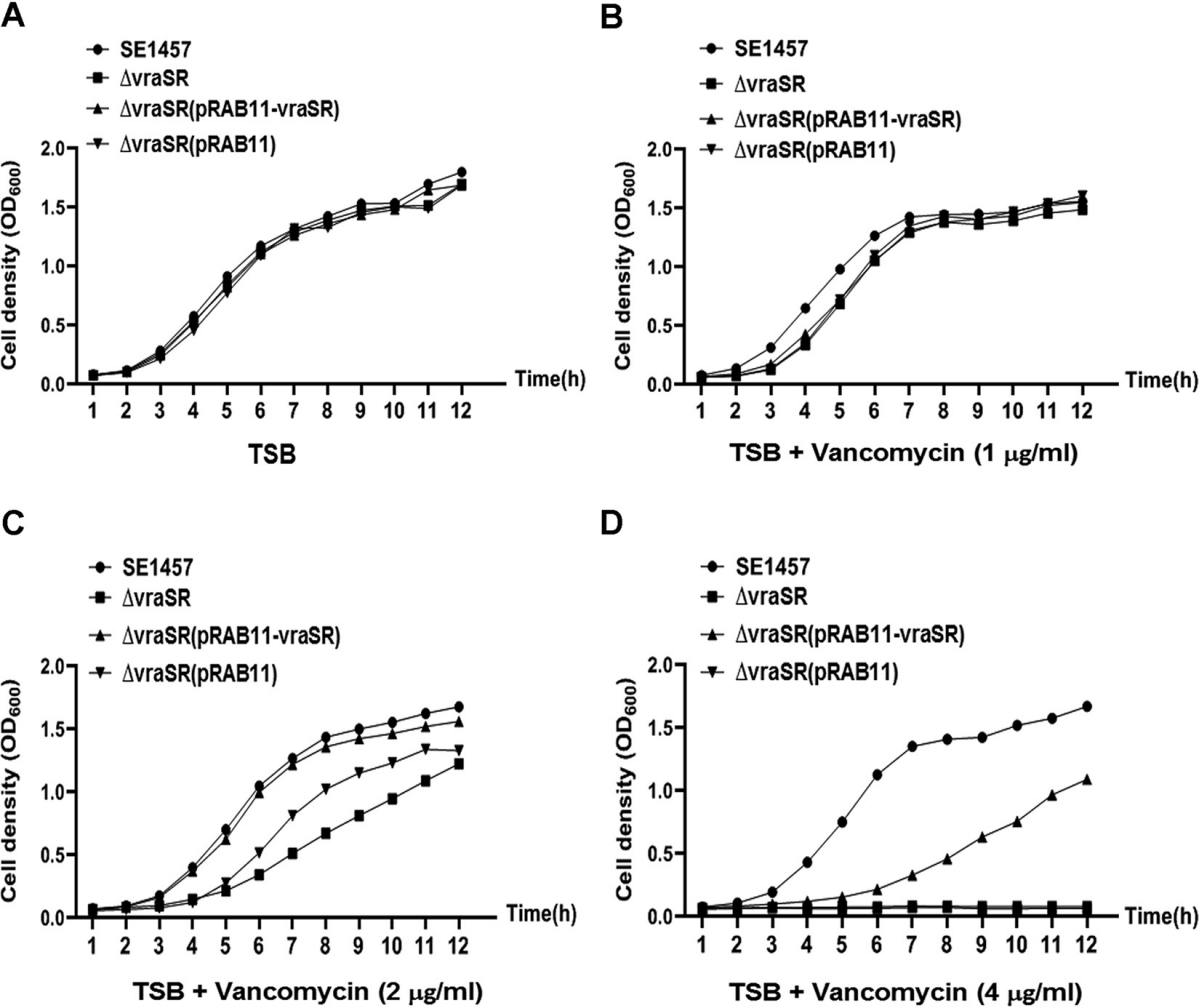
Growth curves of SE1457 isogenic *vraSR* deletion mutants with or without vancomycin. Overnight cultures of SE1457, Δ*vraSR*, Δ*vraSR*(pRAB11-*vraSR*), and Δ*vraSR*(pRAB11) strains were diluted (1:200) in fresh TSB medium with (B, C, and D) or without (A) vancomycin and inoculated into a flask (1:10 culture-to-flask volume ratio) at 37°C with shaking. The cultures were measured hourly at an OD_600_ for 12 h. The curve represents the results of one of the three independent experiments.

Additionally, growth curves of the SE1457 *vraSR* isogenic mutants were determined in tryptone soy broth (TSB) medium with or without vancomycin stress. The deletion of *vraSR* had no effect on the growth of S. epidermidis strain 1457. However, the growth of the Δ*vraSR* mutant cultured in TSB medium with 1/4× or 1/2× MIC of vancomycin (1 or 2 μg/ml) was inhibited slightly compared to that of parent strain SE1457. When the vancomycin stress reached 1× MIC (4 μg/ml), the growth of the Δ*vraSR* and Δ*vraSR*(pRAB11) mutants was inhibited completely, and growth was partly restored in the Δ*vraSR*(pRAB11-*vraSR*) complementation strain (Fig. 2). This result was consistent with that of the susceptibility test.

### Deletion of *vraSR* decreased the tolerance to SDS stress.

**FIG 3:**
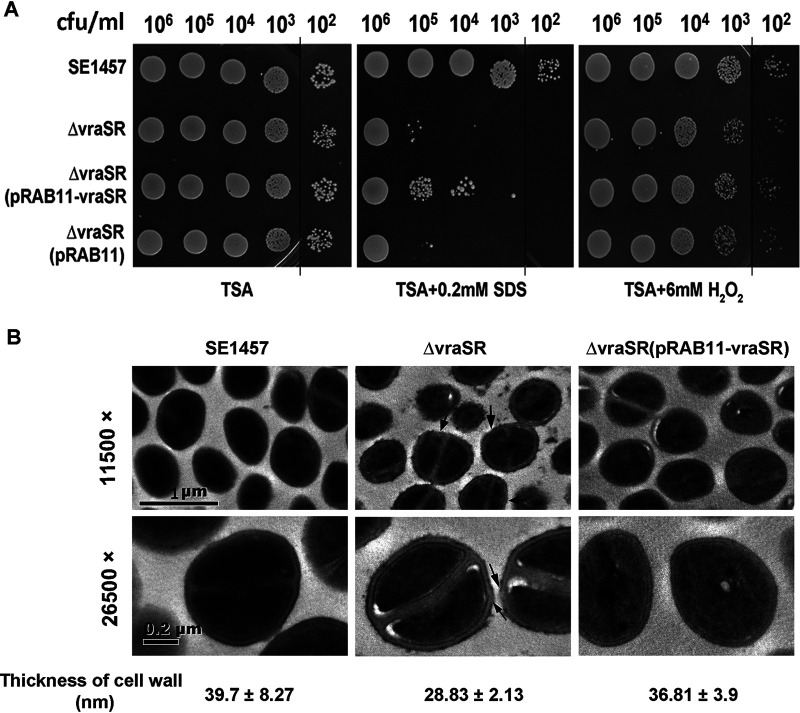
Tolerance of the Δ*vraSR* mutant to SDS and H_2_O_2_. (A) Overnight cultures of S. epidermidis strains were inoculated (1:200) in fresh TSB medium and grown to logarithmic phase (6 h; OD_600_, 2.5) at 37°C. The cultures were serially diluted (1:10), and the aliquot (5 μl) was spotted onto the TSA plate containing 6 mM H_2_O_2_ or 0.2 mM SDS and then incubated at 37°C for 24 h. The colonies on the TSA were photographed. (B) Bacterial morphology of the Δ*vraSR* mutant observed by TEM. The ultrastructure of the log-phase bacteria was observed using TEM. The thickness of the cell wall was measured using Image-Pro Plus 6.0 software and is expressed as the mean ± SD. Arrows indicate the roughness or interruption of the cell wall in the Δ*vraSR* mutant.

To evaluate the effect of *vraSR* deletion on the tolerance of S. epidermidis to stress, bacterial cells with 10-fold serial dilution were spotted on tryptone soy agar (TSA) containing 0.2 mM SDS or 6 mM H_2_O_2_ at 37°C for 24 h of incubation. Compared to parent strain SE1457, the Δ*vraSR* mutant showed a dramatically decreased tolerance (>1,000-fold) to SDS. The Δ*vraSR*(pRAB11-*vraSR*) complementation strain had partially restored tolerance to SDS, while the Δ*vraSR*(pRAB11) vector control displayed a phenotype similar to that of the Δ*vraSR* mutant. Besides, *vraSR* deletion did not affect the tolerance of S. epidermidis to H_2_O_2_ oxidative stress (Fig. 3A).

The morphology of SE1457 and its isogenic *vraSR* mutants was further observed using transmission electron microscopy (TEM). A disrupted and thinner cell wall was observed in the Δ*vraSR* mutant (28.83 ± 2.13 nm) in comparison to parent strain SE1457 (39.7 ± 8.27 nm), and the thickness of the cell wall was not uniform in the Δ*vraSR* mutant, while the thickness of the cell wall was almost restored in the Δ*vraSR*(pRAB11-*vraSR*) complementation strain (36.81 ± 3.9 mm) (Fig. 3B).

### Deletion of *vraSR* impaired biofilm formation *in vitro*.

**FIG 4:**
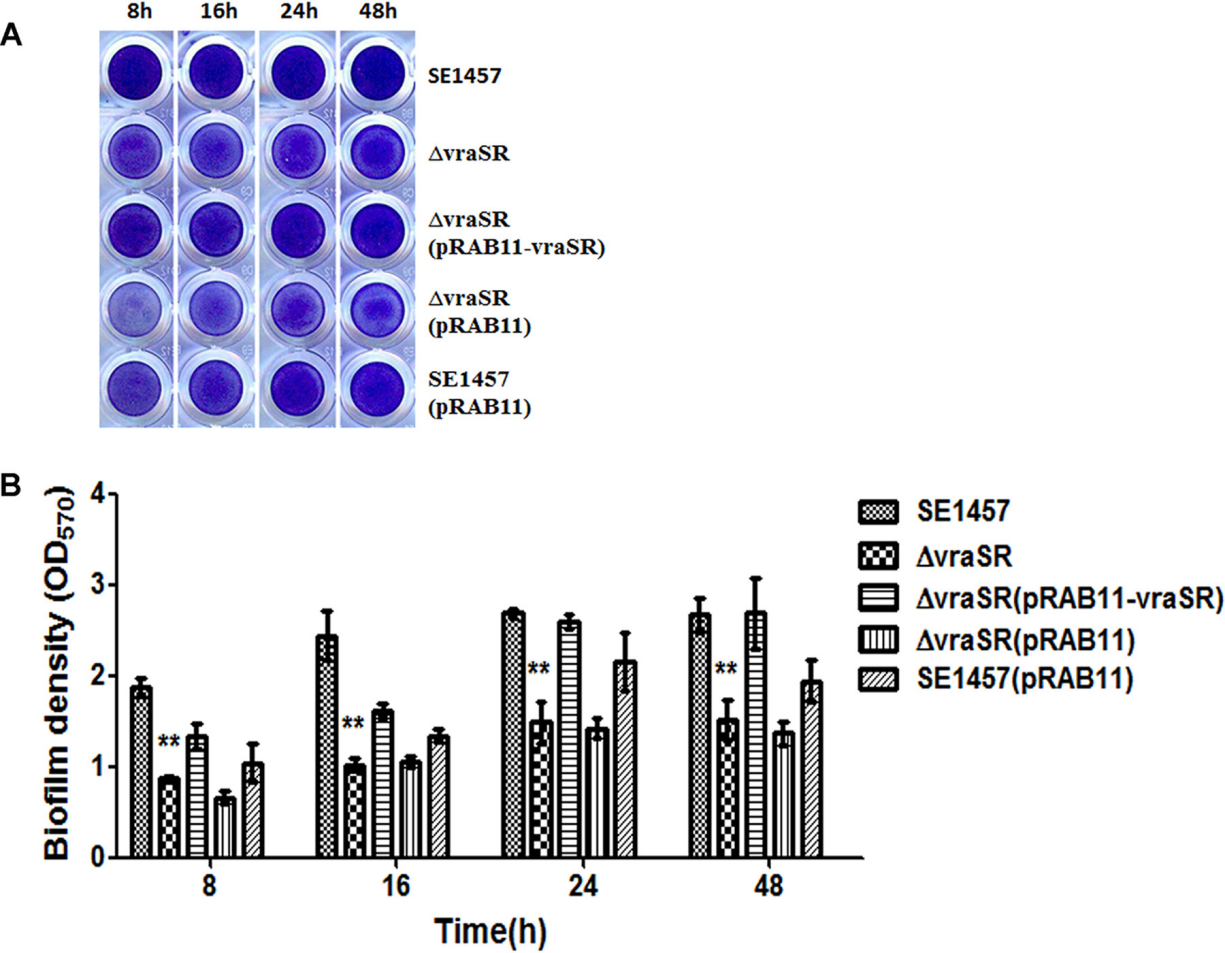
Biofilm formation by the Δ*vraSR* mutant on microtiter plates. Overnight cultures of the S. epidermidis strains were diluted (1:200) with fresh TSB and inoculated into 96-well polystyrene plates in triplicate. After static incubation for 8, 16, 24, and 48 h, biofilms were stained with crystal violet and detected at an OD_570_. The experiments were repeated at least 3 times, and the data represent means ± SD. **, *P < *0.01 (Δ*vraSR* mutant versus SE1457).

The impact of *vraSR* deletion on biofilm formation of S. epidermidis *in vitro* was detected by the polystyrene plate assay and confocal laser scanning microscopy (CLSM). Bacterial biofilm formed on 96-well microtiter plates at 8, 16, 24, and 48 h was stained with crystal violet (Fig. 4A), and biomass was indicated by the optical density at 570 nm (OD_570_) values (Fig. 4B). The Δ*vraSR* mutant produced significantly less biofilm than the parent strain at these time points. For instance, the biofilm formed by the Δ*vraSR* mutant (OD_570_, 1.49 ± 0.23) was dramatically decreased compared to that of wild-type strain SE1457 (OD_570_, 2.68 ± 0.05) at the 24-h time point. Biofilm formation was completely restored in the Δ*vraSR*(pRAB11-*vraSR*) complementation strain (OD_570_, 2.59 ± 0.09) but not in the Δ*vraSR*(pRAB11) control strain (OD_570_, 1.42 ± 0.1).

**FIG 5:**
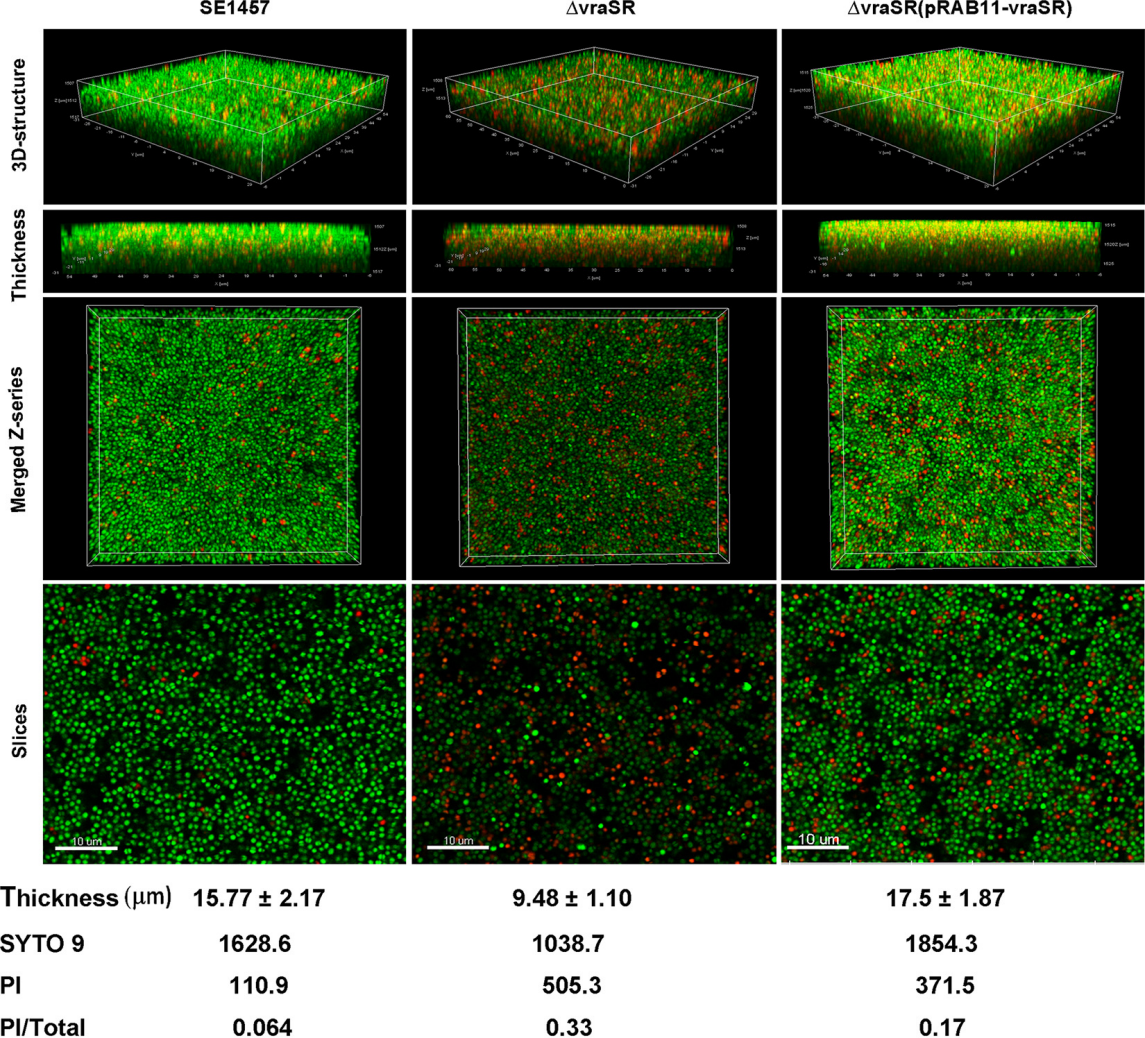
Biofilms of the Δ*vraSR* mutant observed by CLSM. The 24-h biofilms grown on a cover glass in a cell culture dish were visualized using Live/Dead viability staining under a CLSM. Three-dimensional (3-D) structural images (zoom 1, ×63 magnification) were reconstructed, and the thickness of the biofilms was measured using Imaris software. The viable and dead cells were stained in green (SYTO9) and red (PI), respectively. The total amount of fluorescence from the bottom to the top layer of the biofilm was quantified using ImageJ software (zoom 3, ×63 magnification). The PI/total fluorescence value indicates the proportion of dead cells within the biofilm. The figures represent one of three independent experiments.

Furthermore, biofilms of the SE1457, Δ*vraSR*, and Δ*vraSR*(pRAB11-*vraSR*) strains were examined under CLSM with Live/Dead staining. After incubation at 37°C for 24 h, the thickness of the Δ*vraSR* mutant biofilm was much less (9.48 ± 1.10 μm) than that of the parent strain (15.77 ± 2.17 μm) and was restored by complementation with pRAB11-*vraSR* (17.5 ± 1.87 μm). Additionally, the percentage of dead cells in the biofilm of the Δ*vraSR* mutant (33%) was 5-fold higher than that in the biofilm of SE1457 (6.4%), whereas it was decreased in the Δ*vraSR*(pRAB11-*vraSR*) complementation strain (17%) (Fig. 5).

### Deletion of *vraSR* abolished biofilm development *in vivo*.

**FIG 6:**
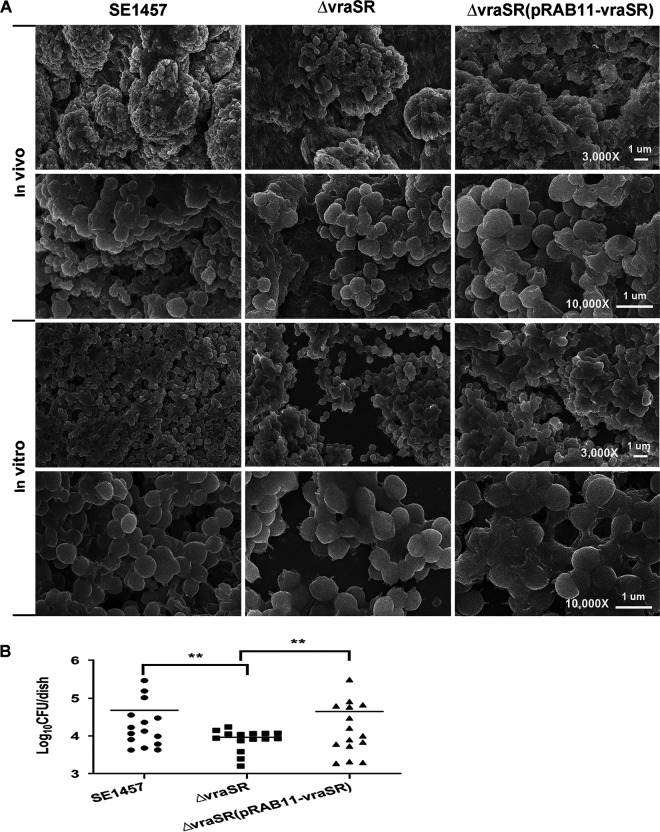
Biofilm formation *in vivo* of the Δ*vraSR* mutant observed under SEM. The New Zealand rabbit model of local S. epidermidis biofilm infection was used. The incisions were subcutaneously made on the back of the animal. Sterile polyethylene disks were implanted, and then overnight cultures (10^8^ CFU) resuspended in 1 ml of TSB were inoculated into the cavities. (A) After 72 h, the disks covered by biofilms were removed, fixed with 2.5% glutaraldehyde, and observed using SEM. As a control, 24-h biofilms cultured in a 6-well plate *in vitro* were observed under SEM. (B) The biofilms formed on the disks were scraped, and the viable bacteria were determined by CFU counting. The data are from one of three independent experiments. **, *P < *0.01 [Δ*vraSR* mutant versus SE1457, Δ*vraSR*(pRAB11-*vraSR*) versus Δ*vraSR* mutant].

To determine whether *vraSR* deletion had an impact on *in vivo* biofilm formation, a subcutaneous foreign body infection model in the rabbit was used. S. epidermidis strains (1 × 10^8^ CFU each) were injected into cavities on the animal’s back where the implanted polystyrene disks were placed. Biofilms that formed on the disks were examined by scanning electron microscopy (SEM) after 72 h. SE1457 formed a compact and thick biofilm covered with the secreted substance on the disks, whereas the Δ*vraSR* mutant formed a much thinner biofilm. The numbers of viable bacterial cells in the Δ*vraSR* biofilm (9.21 × 10^3^ CFU/dish) were lower than those in the SE1457 biofilm (4.82 × 10^4^ CFU/dish) (*P < *0.01) and those in the Δ*vraSR*(pRAB11-*vraSR*) biofilm (4.48 × 10^4^ CFU/dish) (*P < *0.01) (Fig. 6).

### Deletion of *vraSR* affected the biofilm matrix production.

**FIG 7:**
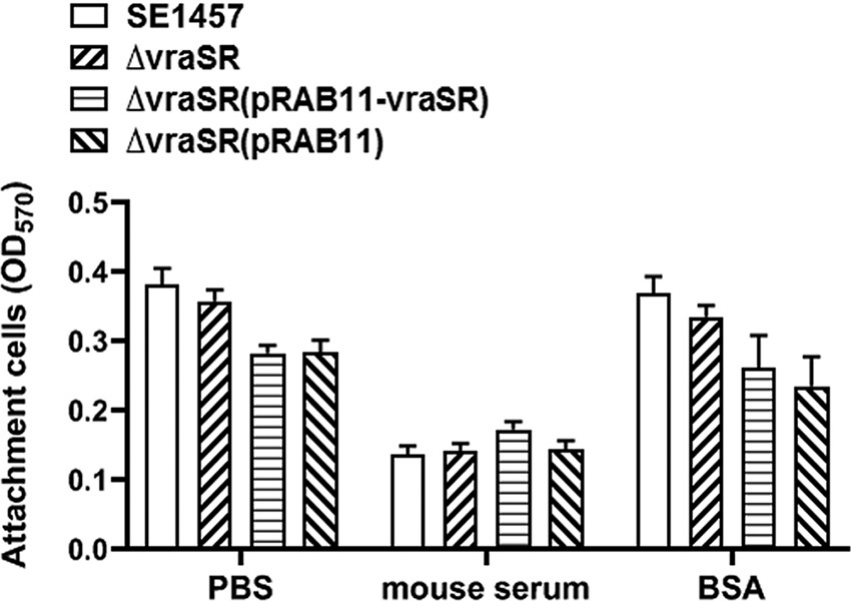
Initial adherence capacity *in vitro* of the Δ*vraSR* mutant. Overnight cultures were diluted (1:200) into fresh TSB medium, and bacteria grown to the log phase (OD_600_, 1.0) were pipetted into the microplate coated with mouse serum or BSA. After 2 h of incubation at 37°C, the plates were washed with PBS and then stained with crystal violet. The initial adherence capacity of the S. epidermidis strains was measured at OD_570_. The results (means ± SD) are from three independent experiments.

Biofilm formation by S. epidermidis is generally a multiple-stage process involving initial bacterial attachment, accumulation, and a subsequent maturation phase. The initial attachment of the Δ*vraSR* mutant to polystyrene plates coated with phosphate-buffered saline (PBS), mouse serum, or bovine serum albumin (BSA) was determined by crystal violet staining with the measurement at OD_570_. In the first stage of biofilm development (the initial attachment of the bacterial cells), no difference was observed between SE1457 and Δ*vraSR* mutant strains (*P > *0.05) (Fig. 7).

**FIG 8:**
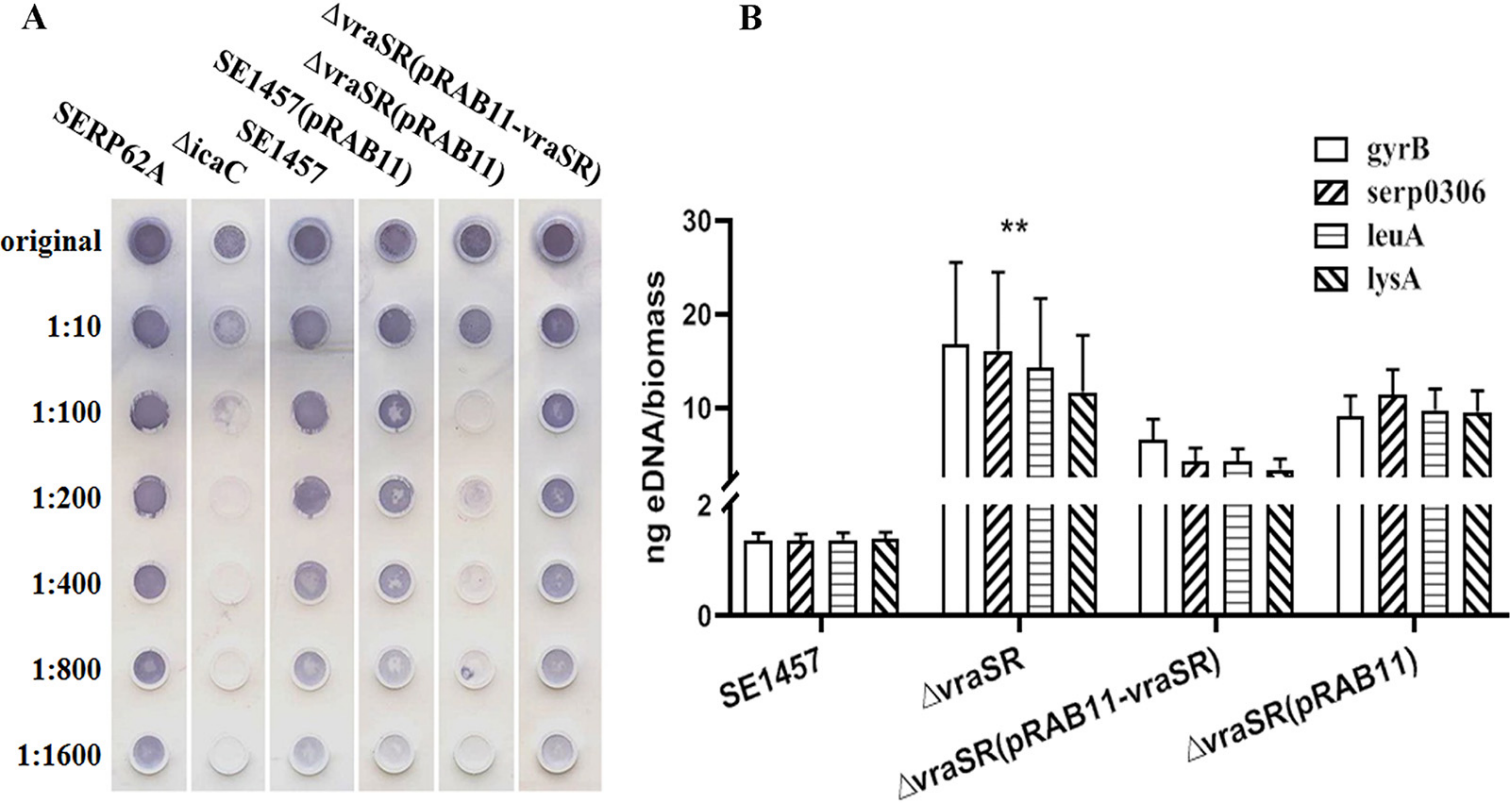
Extracellular matrix biosynthesis of the Δ*vraSR* mutant. (A) PIA biosynthesis was semiquantified by dot blot assay with WGA. The biofilms cultured for 24 h were scraped off and suspended in EDTA. Serial dilutions of PIA extracts were spotted onto nitrocellulose membranes, subsequently incubated with the HRP-conjugated WGA, and then visualized using chromogenic detection. (B) eDNA was quantified by qPCRs of four chromosomal loci (*gyrB*, *serp0306*, *leuA*, and *lysA*). The OD_600_ of unwashed 24-h biofilms was measured to normalize biofilm biomass, and then eDNA was isolated from the biofilms using phenol-chloroform-isoamyl alcohol extraction and ethanol precipitation. The results are shown as the amount of eDNA per biofilm biomass (means ± SD) from three independent experiments.

In the maturation stage of biofilm development, production of the biofilm matrix components including PIA and extracellular DNA (eDNA) was determined in the S. epidermidis strains. PIA, a major factor affecting biofilm maturation and accumulation, was detected semiquantitatively with wheat germ agglutinin-horseradish peroxidase (WGA-HRP). PIA production in the Δ*vraSR*(pRAB11) strain was ∼100-fold less than that in either SE1457 or SE1457(pRAB11) and was restored in the Δ*vraSR*(pRAB11-*vraSR*) complementation strain (Fig. 8A). The relative concentrations of eDNA in 24-h biofilms of the Δ*vraSR* mutant and the Δ*vraSR*(pRAB11) vector control strain were ∼10-fold higher than that of either parent strain SE1457 or the Δ*vraSR*(pRAB11-*vraSR*) complementation strain, as shown in Fig. 8B.

### Comparison of the transcriptomes of the Δ*vraSR* mutant and SE1457.

To compare the transcriptional profile of the Δ*vraSR* mutant with that of SE1457, RNA was extracted from logarithmic-phase bacteria (6 h) in which the expression of *vraSR* was highest (Fig. S2) and detected by transcriptome sequencing (RNA-Seq). The sequencing libraries were prepared in triplicate for the Δ*vraSR* mutant and the parent strain. After removal of ambiguous reads, more than 90% of the reads mapped to S. epidermidis RP62A. A gene with a false discovery rate (FDR)-adjusted *P* value of less than 0.05 (*t* test), a *q* value of less than 0.05, and at least a 2-fold change in transcription level was considered to be differentially expressed. There were 73 genes differentially expressed between the Δ*vraSR* mutant and the SE1457 parent strain; these genes were involved in biofilm formation, bacterial programmed cell death (PCD), glycolysis/gluconeogenesis, the pentose phosphate pathway, and the TCA cycle, etc. Among them, 15 genes were upregulated and 58 genes were downregulated in the Δ*vraSR* mutant. The genes with downregulated expression included the following: the biofilm formation-related gene *icaA* (*N*-acetylglucosaminyltransferase); murein hydrolase modulators *lrgAB* (antiholin-like protein); glycolysis-related genes *gntPKR* and *glpFKD*; pentose phosphate pathway-related genes *sucCD* (succinyl coenzyme A [succinyl-CoA] ligase subunits beta and alpha), *serp2324* (acetoin dehydrogenase), *serp0731* (succinate dehydrogenase), *sdhAB* (l-serine dehydratase), etc.; carbohydrate metabolic process-related genes *manA2* (mannose-6-phosphate isomerase), *malA* (alpha-glucosidase), *rbsKD* (ribokinase), etc.; and phosphate transport-related genes such as *serp2114* (phosphotransferase [PTS] glucose EIICBA component), *serp2343* (PTS mannose transporter subunit IIA), *serp2260* (fructose-specific IIABC components), and *lacF* (PTS lactose-specific phosphotransferase IIA component) (Table 2).

**TABLE 2:** Transcription levels of genes involved in antibiotic tolerance and biofilm formation of S. epidermidis Δ*vraSR*

Gene and locus	GenBank accession no. (location)	Description or predicted function	Expression ratio (mutant/WT)	
			RNA-Seq	qRT-PCR^*a*^
Genes involved in drug tolerance				
*vraR*	NC_002976.3 (1484739–1485368)	DNA-binding response regulator	0.00	0.00001
*vraS*	NC_002976.3 (1485358–1486404)	Two-component sensor histidine kinase	0.00	0.00001
*pbp1*	NC_002976.3 (741901–744228)	Penicillin-binding protein 1	1.32	1.08 ± 0.10
*pbp2*	NC_002976.3 (1063849–1066080)	Penicillin-binding protein 2	1.01	1.06 ± 0.19
*pbp3*	NC_002976.3 (1155781–1157871)	Penicillin-binding protein 3	1.12	ND
*SERP_RS01330*	NC_002976.3 (235619–236599)	Penicillin-V acylase	1.04	ND
*SERP1412(sgtB)*	NC_002976.3 (1476585–1477394)	Monofunctional peptidoglycan glycosyltransferase	0.73	0.85 ± 0.25
*SERP_RS06395*	NC_002976.3 (1340351-1341256)	Transglycosylase	0.95	ND
*murAA*	NC_002976.3 (1747152–1748417)	UDP-*N*-acetylglucosamine-1-carboxyvinyltransferase	0.98	1.08 ± 0.13
*murE*	NC_002976.3 (600125–601609)	UDP-*N*-acetylmuramoyl-l-alanyl-d-glutamate–l-lysine ligase	1.17	0.99 ± 0.20
*pflA*	NC_002976.3 (2411378–2412133)	Pyruvate formate lyase activating enzyme	0.30	0.77 ± 0.23
*plfB*	NC_002976.3 (2412155-2414401)	Formate C-acetyltransferase	0.41	ND
*lrgA*	NC_002976.3 (2047084–2047482)	Antiholin-like murein hydrolase modulator LrgA	0.09	0.10 ± 0.11
*lrgB*	NC_002976.3 (2047486–2048187)	Antiholin-like protein LrgB	0.16	ND
*cidA*	NC_002976.3 (2142373–2142765)	Holin-like protein, CidA/LrgA family protein	2.20	4.60 ± 0.46
				
Genes involved in biofilm formation				
*icaA*	NC_002976.3 (2334220–2335458)	Poly-beta-1,6-*N*-acetyl-d-glucosamine synthase	0.55	0.17 ± 0.05
*icaR*	NC_002976.3 (2333498–2334055)	*icaADBC* negative transcriptional regulator	0.89	9.27 ± 4.4
*atlE*	NC_002976.3 (627656-631663)	Bifunctional autolysin	0.88	0.89 ± 0.10
*rsbU*	NC_002976.3 (1724457–1725458)	Serine/threonine phosphatase	1.48	0.84 ± 0.13
*sarA*	NC_002976.3 (279424–279798)	Staphylococcal accessory regulator A	0.88	0.89 ± 0.18
*aap*	NC_002976.3 (2459164–2460687)	Accumulation-associated protein	0.98	0.77 ± 0.12

Genes involved in glycolysis/gluconeogenesis				
*gntP*	NC_002976.3 (2081933–2083291)	Gluconate transporter	0.21	ND
*gntK*	NC_002976.3 (2083341–2084882)	Gluconate kinase	0.16	0.34 ± 0.19
*gntR*	NC_002976.3 (2084906–2085586)	GntR family transcriptional regulator	0.43	ND
*SERP_RS10290*	NC_002976.3 (2094468–2095742)	Glucarate transporter, MFS transporter	0.33	ND
*SERP_RS11465*	NC_002976.3 (2371584–2372924)	Sugar porter family MFS transporter	0.28	ND
*SERP_RS10325*	NC_002976.3 (2100973–2102937)	Fructose-1,6-bisphosphatase	0.47	ND
*glpF*	NC_002976.3 (879638–880462)	Glycerol transporter, aquaporin family protein	0.22	ND
*glpK*	NC_002976.3 (880616–882115)	Glycerol kinase	0.20	ND
*glpD*	NC_002976.3 (882292–883965)	Aerobic glycerol-3-phosphate dehydrogenase/oxidase	0.09	0.38 ± 0.11
*larB*	NC_002976.3 (261972–262748)	1,5-Phosphoribosyl-5-amino-4-imidazole carboxylate carboxylase	0.43	ND
*larE*	NC_002976.3 (262763–263590)	ATP-dependent sacrificial sulfur transferase	0.49	ND
*SERP_RS01465*	NC_002976.3 (263610–264878)	Nickel-dependent lactate racemase	0.42	ND
*adhP*	NC_002976.3 (265167–266189)	Zinc-dependent alcohol dehydrogenase	0.43	ND
*SERP_RS01475*	NC_002976.3 (266272–266664)	Hypothetical protein	0.43	ND
*pckA*	NC_002976.3 (1409889–1411481)	Phosphoenolpyruvate carboxykinase (ATP)	0.36	ND
*SERP_RS01910*	NC_002976.3 (355256–356014)	DeoR/GlpR family transcriptional regulator	5.39	ND
*fruK*	NC_002976.3 (356011–356931)	1-Phosphofructokinase	8.53	11.43 ± 2.99
*fruA*	NC_002976.3 (356937–358889)	PTS fructose transporter subunit IIC	5.91	ND
*pfkA*	NC_002976.3 (1303827–1304795)	ATP-dependent 6-phosphofructokinase	2.88	ND
*gapR*	NC_002976.3 (445408–446421)	Sugar-binding transcriptional regulator	4.59	ND
*gapA-1*	NC_002976.3 (446473–447483)	Type I glyceraldehyde-3-phosphate dehydrogenase	2.67	ND
*pgk*	NC_002976.3 (447677–448867)	Phosphoglycerate kinase	2.99	ND
*pyk*	NC_002976.3 (1302046–1303803)	Pyruvate kinase	2.54	ND

Genes involved in pentose phosphate pathway				
*sucC*	NC_002976.3 (815834–817000)	ADP-forming succinate-CoA ligase subunit beta	0.46	0.31 ± 0.18
*sucD*	NC_002976.3 (817022–817930)	Succinyl-CoA ligase subunit alpha	0.38	ND
*SERP_RS03700*	NC_002976.3 (730806–732572)	Succinate dehydrogenase, flavoprotein subunit	0.45	ND
*SERP_RS03705*	NC_002976.3 (732572–733414)	Succinate dehydrogenase iron-sulfur subunit	0.39	ND
*SERP_RS04930*	NC_002976.3 (1001204–1002466)	Dihydrolipoyl lysine residue succinyltransferase component of 2-oxoglutarate dehydrogenase complex	0.21	ND
*SERP_RS04935*	NC_002976.3 (1002485–1005289)	2-Oxoglutarate dehydrogenase subunit E1 component	0.28	ND
*SERP_RS11420*	NC_002976.3 (2360269–2361546)	Acetoin dehydrogenase subunit E2	0.38	0.11 ± 0.08
*SERP_RS11425*	NC_002976.3 (2361560–2362600)	Acetoin dehydrogenase, E1 component, beta subunit	0.16	ND
*SERP_RS11430*	NC_002976.3 (2362671–2363624)	Thiamine pyrophosphate-dependent dehydrogenase E1 component subunit alpha	0.35	ND
*sdhA*	NC_002976.3 (2118833–2119732)	l-Serine dehydratase, iron-sulfur-dependent, subunit alpha	0.31	0.65 ± 0.13
*sdhB*	NC_002976.3 (2119745–2120425)	l-Serine ammonia-lyase, iron-sulfur-dependent, subunit beta	0.46	ND
*mqo-3*	NC_002976.3 (2350001–2351497)	Malate quinone oxidoreductase	2.29	ND
*icd*	NC_002976.3 (1296195–1297463)	NADP-dependent isocitrate dehydrogenase	0.43	ND
*gltA*	NC_002976.3 (1297506–1298627)	Citrate synthase, catalyzes the formation of citrate from acetyl-CoA and oxaloacetate	0.45	ND

Genes involved in phosphate transport system				
*SERP_RS10495*	NC_002976.3 (2137627–2139654)	PTS glucose EIICBA component	0.14	0.48 ± 0.01
*SERP_RS11510*	NC_002976.3 (2389604–2389978)	PTS mannose transporter subunit IIA	0.41	ND
*SERP_RS11515*	NC_002976.3 (2389982–2390557)	Dihydroxyacetone kinase subunit L	0.46	ND
*SERP_RS11135*	NC_002976.3 (2289420–2291360)	PTS system, fructose-specific IIABC components	0.27	ND
*SERP_RS09555*	NC_002976.3 (1928396–1929985)	PTS alpha-glucoside transporter subunit IIBC	0.17	ND
*lacF*	NC_002976.3 (1838149–1838463)	PTS system lactose-specific phosphotransferase IIA component	0.40	ND
*lacD*	NC_002976.3 (1838483–1839460)	Tagatose 1,6-diphosphate aldolase	0.42	ND
*SERP_RS11820*	NC_002976.3 (2467765–2469144)	Hexose phosphate transporter, phosphoglycerate transporter family protein	0.31	ND
*manA-2*	NC_002976.3 (2291376–2292326)	Mannose-6-phosphate isomerase class I	0.11	0.14 ± 0.01
*malA*	NC_002976.3 (1114441–1116096)	Alpha-glucosidase	0.44	ND
*rbsK*	NC_002976.3 (2124965–2125888)	Ribokinase	0.17	ND
*rbsD*	NC_002976.3 (2125942–2126346)	d-Ribose pyranase	0.17	ND
*rbsU*	NC_002976.3 (2126368–2127249)	Ribose transporter, membrane protein	0.19	ND
*SERP_RS05175*	NC_002976.3 (1083213–1083662)	Nucleoside diphosphate kinase	0.39	ND

Genes involved in amino acid metabolism				
*rocD*	NC_002976.3 (529033–530223)	Ornithine oxoacid transaminase	0.49	ND
*gluD*	NC_002976.3 (530332–531576)	Glutamate dehydrogenase, NAD specific	0.46	ND
*putA*	NC_002976.3 (1385177–1386178)	Proline-dehydrogenase	0.46	ND
*SERP_RS10565*	NC_002976.3 (2152253–2153797)	l-Glutamate gamma-semialdehyde dehydrogenase	0.42	ND
*geh-1*	NC_002976.3 (2337843–2339909)	Lipase, YSIRK domain-containing triacylglycerol lipase GehC	0.37	ND
*arcC*	NC_002976.3 (2397500–2398432)	Carbamate kinase	0.27	ND
*SERP_RS11560*	NC_002976.3 (2398606–2400162)	Membrane protein, YfcC family protein	0.34	ND

a qRT-PCR data are given as the means ± standard deviations of the results from three independent experiment. ND, not done.

10.1128/mSphere.00641-21.2 FIG S2 Transcriptional analysis of SE1457 *vraS* and *vraR* in different growth phases. RNA samples were isolated from SE1457 grown in the same flask at early (4 h), mid (6 h), and late (8 h) log phase, and stationary phase (10 h and 12 h). The levels of *vraS* and *vraR* transcripts were normalized against the level of the *gyrB* transcript. Data represented the mean ± SD of results from three independent experiments. Download FIG S2, TIF file, 0.2 MB. Copyright © 2021 Wu et al. 2021 Wu et al. https://creativecommons.org/licenses/by/4.0/ This content is distributed under the terms of the Creative Commons Attribution 4.0 International license.

The altered expression of the genes mentioned above was confirmed by qRT-PCR. It showed that the transcriptional level of *icaA* was downregulated about 6-fold in the Δ*vraSR* mutant, while the mRNA level of *icaR* was upregulated about 9-fold, in comparison with those in SE1457. In addition, corresponding with a 5-fold upregulation of *cidA* (holin-like protein) expression, *lrgA* (antiholin-like protein) transcription was downregulated about 10-fold. However, the transcriptional levels of drug-resistant genes, such as *pbp2*, *serp1412* (*sgtB* analog), and *murAA*, etc., showed no significant changes.

### Binding of recombinant VraR protein to the *ica* promoter region.

**FIG 9:**
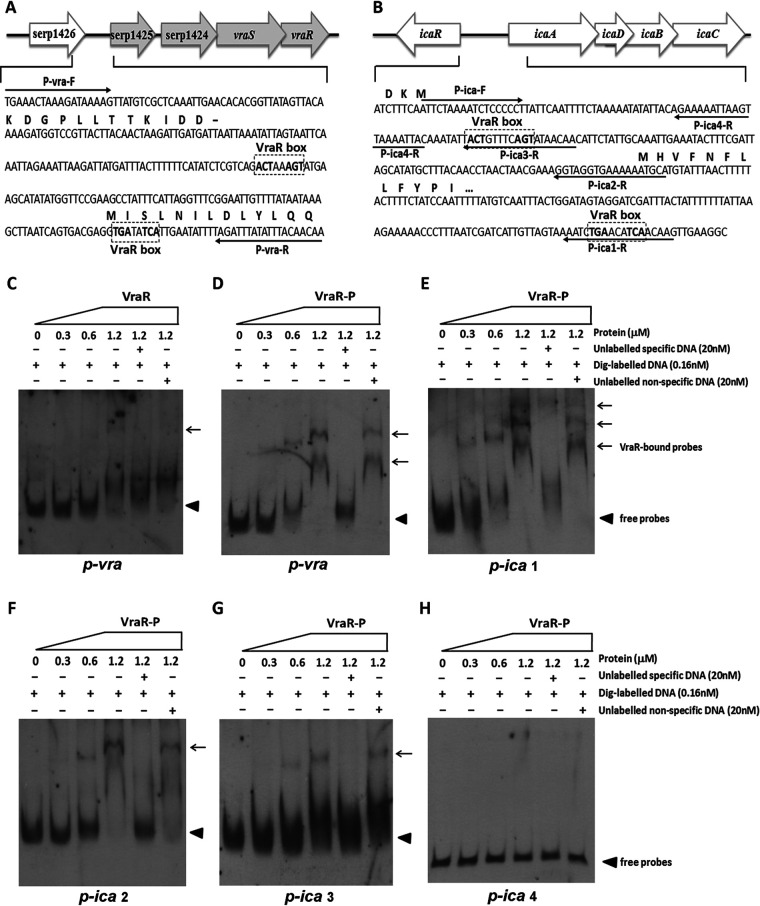
EMSA analysis of S. epidermidis VraR with the putative promoter regions. His-tagged VraR was purified and phosphorylated (VraR-P) by incubation with 50 mM acetyl phosphate. The putative promoter regions of *vraSR*, *ica*, *pbp2*, *sgtB*, and *murAA* genes were PCR amplified. DNA probes were labeled with digoxigenin (Dig). Electrophoretic mobility shift assays (EMSAs) were performed by incubating labeled probes with increasing amounts of VraR-P (range, 0.3 to 1.2 μM). For each blot, lane 1 contained a no-protein control and lanes 5 and 6 contained a 125-fold excess of the unlabeled specific probe (competitor control) and unlabeled nonspecific probe (DNA fragment within the *rpsJ* coding region), respectively. Reaction mixtures were incubated for 20 min at 25°C, separated in a nondenaturing polyacrylamide gel (6%), and then blotted onto a nylon membrane. After incubation with anti-digoxigenin antibody, CSPD chemiluminescent reagent was added. Triangles indicate the positions of free probes; arrows indicate the positions of the VraR-DNA complex.

The VraR binding box was reported to have the pattern ACT(X)nAGT or TGA(X)nTCA (n = 1 to 3 nucleotides [nt]) in S. aureus. Bioinformatics analysis showed that similar VraR boxes were present in the upstream region of *vra* and *ica* operons in the genome of S. epidermidis RP62A (Fig. 9A and B). To further study the regulatory role of VraSR in biofilm formation, an EMSA was carried out to detect the binding of recombinant VraR (His-tagged VraR) to the putative promoter regions labeled with digoxigenin. The 281-bp DNA fragment upstream of *vraSR* (p-*vra*) formed a shifted complex with phosphorylated VraR (VraR-P) in a dose-dependent manner but did not form such a complex with unphosphorylated VraR (Fig. 9C and D, lanes 2 to 4). The addition of a 125-fold excess of unlabeled p-*vra* as a specific competitor blocked VraR-DNA-probe complex formation (lane 5), while the same amount of the unlabeled nonspecific DNA (119-bp fragment of *rpsJ* coding region) as a control did not affect the shifted complex formation (lane 6).

Furthermore, VraR-P resulted in mobility shifts of the 301-bp fragment (p-*ica* 1) containing two putative VraR boxes (Fig. 9E) and bound to the 170-bp fragment (p-*ica* 2) and 92-bp fragment (p-*ica* 3) containing only one VraR box (Fig. 9F and G) but did not bind to the 66-bp fragment (p-*ica* 4) that lacked the putative VraR box (Fig. 9H). The results indicated that VraR-P was able to bind specifically to the promoter regions of the *ica* operon as well as its own. VraR-P did not bind to the fragment upstream of *pbp2*, *serp1412*, or *murAA* (data not shown).

## DISCUSSION

VraSR in S. aureus has been previously recognized as a resistance-associated regulatory system that regulates the transcription of genes (such as *pbp2*, *sgtB*, and *murZ*, etc.) involved in peptidoglycan biosynthesis and influences the susceptibility of S. aureus cells to antibiotics inhibiting cell wall synthesis (10, 15). S. aureus VraSR also modulates the production of virulence factors by binding the P2-P3 intergenic region of the *agr* promoter (6). This study has suggested a different role for VraSR in S. epidermidis biofilm development, stress tolerance, and cell wall homeostasis.

In this work, we demonstrated for the first time that S. epidermidis VraSR was autoregulated. In the genome of S. epidermidis RP62A, *vraSR* forms a four-gene operon together with two upstream genes (*serp1424*, *serp1425*). The loci of *serp1424* and *serp1423* (*vraS*) and of *serp1423* and *serp1422* (*vraR*) overlapped by 4 and 11 nt, respectively. However, the loci of *serp1425* and *serp1424* were separated by 14 nt. There exists a putative promoter region upstream of *serp1425* and a transcription terminator structure (**GGCGAAAGTA**AAGATACATCTATCGA**TACTTTCGCC**; where boldface nucleotides represent reverse complement sequences to form a hairpin structure) located 10 bp downstream of the *serp1422* translational stop codon. The cotranscription of these four genes in a single mRNA was verified by RT-PCR (see Fig. S3 in the supplemental material), and S. epidermidis VraSR autoregulation was further confirmed using an EMSA in which phosphorylated VraR bound to the promoter region of the *vraSR* operon.

10.1128/mSphere.00641-21.3 FIG S3 Cotranscription analysis of the four genes *serp1421* to *serp1424*. (A) Physical map of *vraSR* located in the genome of S. epidermidis RP62a. The *vraR* and *vraS* genes corresponded to the *serp1422* and *serp1423* loci, respectively. Loci of *serp1424* and *serp1425* are located upstream of *vraSR*. The short hairpin structure downstream of *vraR* was the transcriptional terminator of the *vra* operon. Black arrows indicate the positions of primers used for reverse transcription (RT)-PCR amplification. (B) Cotranscription of *vraSR* genes was performed using RT-PCR analysis with cDNA, genomic DNA (gDNA), or RNA as the templates. Lanes 1, 2, and 3 represent the amplification using primers V45-F/V45-R; lanes 4, 5, and 6 represent the amplification using primers V34-F/V34-R; lanes 7, 8, and 9 represent the amplification using primers V23-F/V23-R; lanes 10, 11, and 12 represent the amplification using primers V12-F/V12-R. The expected size of each PCR product is indicated. Download FIG S3, TIF file, 0.5 MB. Copyright © 2021 Wu et al. 2021 Wu et al. https://creativecommons.org/licenses/by/4.0/ This content is distributed under the terms of the Creative Commons Attribution 4.0 International license.

We then found that the expression of the S. epidermidis VraSR system was dependent on the types and concentration of the tested stressors. The *vraS* and *vraR* genes of SE1457 were induced by the cell wall-active agents (vancomycin, ampicillin, or SDS) with an exposure time of 30 min but not by the other stresses, such as chloramphenicol, oxidative stress (H_2_O_2_), hyperosmosis (NaCl), heat, and hypoxia, with the same exposure time (Fig. 1). This indicated that S. epidermidis VraSR selectively responds to cell wall-active antibiotics, which is in accordance with the findings in S. aureus (10, 26).

To study the role of VraSR in regulating biofilm formation of S. epidermidis, a *vraSR* deletion mutant (Δ*vraSR*) from SE1457 was constructed. The deletion of *vraSR* led to impaired biofilm formation both *in vitro* and *in vivo* and to an increased percentage of dead cells within the biofilm (Fig. 4 and 6). The impaired biofilm was restored completely by introducing plasmid pRAB11 containing the *vraSR* system into the Δ*vraSR* mutant strain, and complementation of the empty plasmid control had no effect on the phenotype of the *vraSR* deletion mutant, which indicated that the VraSR system may directly modulate S. epidermidis biofilm formation. However, the increases in dead cells and eDNA release were partly restored in the Δ*vraSR*(pRAB11-*vraSR*) complementation strain, which indicated that there were other regulators participating in cell death and eDNA release.

Previous studies indicated that the Δ*vraSR* strain reduced the ability of S. aureus to survive within PMNs due to decreased biofilm formation (7). However, the exact mechanism by which VraSR modulates staphylococcal biofilm formation is unknown. In the step of initial attachment, the adherence capacity of the Δ*vraSR* mutant to a polystyrene surface precoated with mouse serum or BSA was similar to the level of SE1457 (Fig. 7), which indicated that S. epidermidis VraSR was not directly involved in the initial attachment step of biofilm development. We then further analyzed the production of biofilm matrix in SE1457 isogenic *vraSR* mutant strains (Fig. 8). PIA production, as the most important intercellular adherence factor in the accumulation step of biofilm formation in staphylococci, was decreased more than 100-fold in the Δ*vraSR* mutant compared with that of parent strain SE1457. Extracellular DNA (eDNA) is usually released following bacterial cell death. The amount of eDNA within the Δ*vraSR* mutant biofilm was more than that in the parent strain, which was in accordance with a much higher percentage of dead cells in Δ*vraSR* biofilm, although there was no difference in Triton X-100-induced autolysis between the Δ*vraSR* and SE1457 strains (27) (Fig. S4). These results indicated that S. epidermidis VraSR modulated biofilm formation mainly through interference with PIA production.

10.1128/mSphere.00641-21.4 FIG S4 Autolysis of the Δ*vraSR* mutant induced by Triton X-100. Bacterial cells grown to an OD_600_ of 0.6 to 0.8 in TSB containing 1 M NaCl were washed with ice-cold water, resuspended in 30 ml buffer (0.05 M Tris-HCl, pH 7.2) supplemented with 0.005% Triton X-100, and then incubated at 30°C with shaking. The OD_600_ value was detected at 30-min intervals. The *atlE* deletion mutant derived from SE1457 (Δ*atlE*) acted as a negative control. Download FIG S4, TIF file, 0.3 MB. Copyright © 2021 Wu et al. 2021 Wu et al. https://creativecommons.org/licenses/by/4.0/ This content is distributed under the terms of the Creative Commons Attribution 4.0 International license.

PIA is synthesized by *ica* operon gene products, and the *ica* operon is composed of four genes (*icaADBC*) and a divergently transcribed repressor (*icaR*). A transcriptional profile and qRT-PCR demonstrated that the expression of *icaR* in the Δ*vraSR* mutant was upregulated compared with that in the parent strain and *icaA* was downregulated, which was consistent with the results of the decreased PIA production in the Δ*vraSR* mutant strain.

Studies (8) have found that the VraR-specific binding motif in S. aureus was identified as the sequence 5-ACT(X)nAGT-3 or 5-TGA(X)nTCA-3, where X is any nucleotide and n (number of nucleotides) may vary from 1 to 3. According to the VraR binding motif of S. aureus, we conducted a manual search in the putative promoters of the genes in the genome of S. epidermidis RP62A (Table S2). The putative promoter region upstream of the *ica* operon, drug resistance-related genes (such as *vraSR*, *murAA*, *serp1412*), bacterial cell death-related genes *lrgAB*, and other genes (*serp0331*, *serp0707*) all harbored the VraR binding motif, but this motif was absent in the promoter region of *pbp2* and *atlE* genes.

10.1128/mSphere.00641-21.6 TABLE S2 Analysis of putative promoter regions of genes regulated by VraR. Download Table S2, DOCX file, 0.02 MB. Copyright © 2021 Wu et al. 2021 Wu et al. https://creativecommons.org/licenses/by/4.0/ This content is distributed under the terms of the Creative Commons Attribution 4.0 International license.

We further explored the interaction between VraR and its putative promoter regions (Fig. 9). An EMSA showed that phosphorylated VraR bound to the promoter regions of p*-ica* 1, p*-ica* 2, and p*-ica* 3, respectively, but not to the p*-ica* 4 region. The VraR also bound to its own promoter region, p*-vra*. First, we found that S. epidermidis VraSR directly modulated biofilm formation in an *ica*-dependent manner.

Besides its effect on biofilm formation, VraSR also regulates S. aureus drug resistance. Studies (28, 29) have reported that the *vraSR* deletion mutant strain derived from S. aureus strain N315 exhibited a reduced transcription level of genes associated with cell wall biosynthesis (such as *pbp2*, *sgtB*, and *murZ*), which correlated well with the increased susceptibility of S. aureus to the cell wall synthesis-inhibiting antibiotics (such as glycopeptides and β-lactam). In the present study, a similar drug-susceptible phenotype was observed in the S. epidermidis *vraSR* deletion mutant (Table 1 and Fig. 2; Table S1). The Δ*vraSR* mutant strain had increased susceptibility to vancomycin, ampicillin, cefuroxime, and cefotaxime, in comparison with parent strain SE1457, but not significantly to amikacin, gentamicin, tetracycline, and levofloxacin, as determined by both the broth dilution method and disk diffusion test. The results of both RNA-Seq and qRT-PCR revealed that transcriptional levels of genes related to drug resistance (such as *pbp2*, *serp1412*, and *murAA*, etc.) had no significant change in the Δ*vraSR* mutant strain, and an EMSA demonstrated that the phosphorylated VraR protein failed to bind to the promoter regions of the *pbp2*, *serp1412*, *and murAA* genes, respectively. These results indicated that unlike that of S. aureus, VraSR of S. epidermidis indirectly modulates drug susceptibility upon the environmental stress.

It has been reported that CidA and LrgA represent a holin-antiholin system, which may serve as molecular control elements of bacterial programmed cell death (PCD) (30–32). CidA oligomerizes and forms pores in the cytoplasmic membrane, leading to membrane depolarization and activation of murein hydrolase activity and promoting susceptibility to penicillin, whereas LrgA opposes the activity of CidA by interfering with its ability to depolarize the membrane. The balance between CidA and LrgA is thought to determine bacterial viability (33, 34), and several factors were reported to regulate the expression of the CidA-LrgAB system, such as TCS-SrrAB, LytSR, and CidR, etc. (30, 35, 36). In this study, both RNA-Seq and qRT-PCR also showed that the expression of *cidA* in the *vraSR* mutant strain was upregulated compared with that of parent strain SE1457, and *lrgA* was downregulated (Table 2), which led to more dead cells and higher eDNA release in the Δ*vraSR* mutant. However, complementation of *vraSR* in the Δ*vraSR* mutant could not completely restore the dead cells and eDNA release to the level of parent strain SE1457, which indicated that S. epidermidis VraSR may play a partial role in CidA-LrgAB mediation of cell death. Meanwhile, the Δ*vraSR* mutant strain exhibited thinner and interrupted cell walls compared to parent strain SE1457 under TEM, and the intracellular electron density decreased and cells became swollen in the Δ*vraSR* mutant strain. These structural and morphological changes indicated that *vraSR* deletion interfered with cell wall synthesis or perturbed the cell membrane of S. epidermidis, which could explain why the Δ*vraSR* mutant was more susceptible to cell wall target antibiotics and SDS than strain SE1457 (Fig. 2 and 3), but this deletion had little effect on other agents. These results indicate that S. epidermidis VraSR may influence susceptibility through modulation of cell death by positive regulation of LrgAB expression.

That the increased amount of eDNA could not restore biofilm formation in the *vraSR* deletion mutant indicated that PIA probably plays a more important role in the biofilm formation of S. epidermidis. The role of matrix in biofilm formation of S. epidermidis needs to be further explored.

Additionally, teichoic acids, special components in the cell wall of most Gram-positive bacteria, are bacterial copolymers of glycerol phosphate or ribitol phosphate and carbohydrates linked via phosphodiester bonds and are essential products of glycolysis or the pentose phosphate pathway (PPP) (23, 37). The expression of genes related to glycolysis (*gntPKR*, *glpFKD*), PPP (*serp2324*, *serp2325*, *sucCD*, *serp0731*, *serp0732*), the phosphate transport system (*serp2114*, *serp2343*, *serp2344*, *lacFD*), and the carbohydrate metabolic process (*rbsKDU*, *gluD*, *manA2*) were downregulated in the *vraSR* mutant strain in comparison with parent strain SE1457. The interrupted cell wall or high permeability of the Δ*vraSR* mutant strain may be partially attributed to the down-transcription of the above-mentioned genes, which facilitated the killing of this strain by antibiotics and SDS.

**FIG 10:**
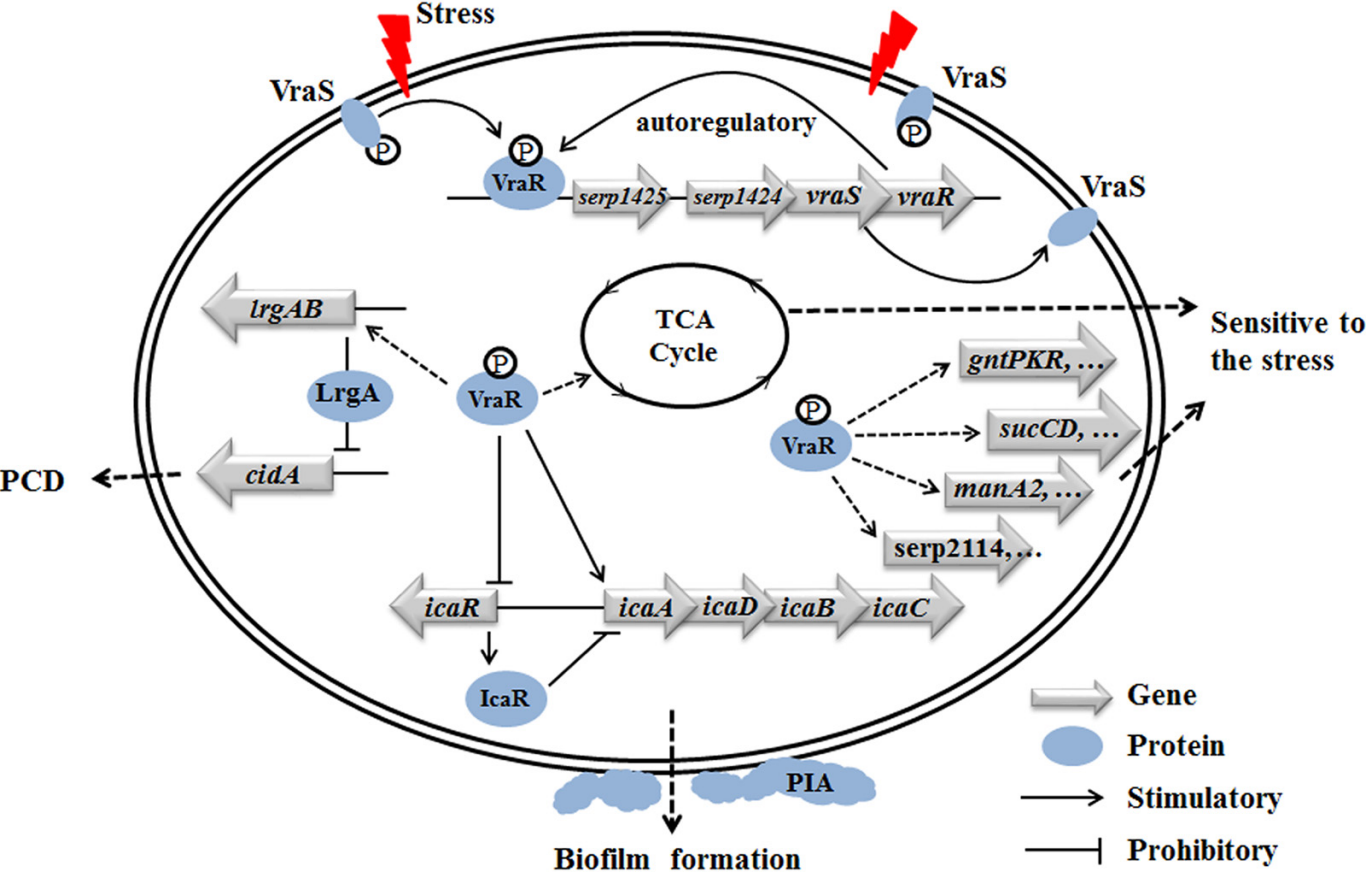
Proposed model of VraSR regulation in S. epidermidis. VraS represents the membrane-associated sensor kinase that becomes activated and autophosphorylated (indicated by a circled “P”) upon the cell wall/membrane damage (indicated by a red flash). The VraS-P phosphorylates VraR to VraR-P, which acts as a response regulator that directly regulates its own *vraSR* operon, as well as the *icaADBC* operon (solid lines). At the same time, VraR-P acts as a repressor for *icaR*, which encodes the repressor of the *ica* operon. Genes that are indirectly positively regulated are indicated by dotted lines.

In summary, S. epidermidis VraSR is autoregulated upon damage to the cell wall structure and directly modulates biofilm formation in an *ica*-dependent manner (Fig. 10). The mechanism by which VraSR influences bacterial susceptibility and cell death may be by regulating the transcription levels of the CidA-LrgAB system and the genes involved in cell wall biosynthesis.

## MATERIALS AND METHODS

### Bacterial strains, plasmids, and growth conditions.

The bacterial strains and plasmids used in this study are listed in Table 3. S. epidermidis 1457 (SE1457) and S. aureus RN4220 were kindly provided by Gao Fu from Hong Kong University. S. epidermidis RP62A (a biofilm-positive strain, accession number NC_002976) (38) was purchased from the American Type Culture Collection (ATCC, Manassas, VA, USA). The temperature-sensitive plasmid pKOR1 was kindly gifted by Li Ming from Fudan University. All staphylococci were routinely cultured in tryptone soy broth (TSB; Oxoid, Basingstoke, UK) or tryptone soy agar (TSA). For detection of biofilm formation, S. epidermidis was cultured in TSB medium supplemented with 0.5% glucose. B2 medium (1% casein hydrolysate, 2.5% yeast extract, 0.5% glucose, 2.5% NaCl, 0.1% K_2_HPO_4_, pH 7.5) was used for the recovery of staphylococcal cells after electroporation. Luria-Bertani (LB) medium was used for the culture of Escherichia coli. When appropriate, antibiotics were used at concentrations of 5 μg/ml for erythromycin (Em), 10 μg/ml for chloramphenicol (Cm), 100 μg/ml for ampicillin (Am), and 50 μg/ml for kanamycin (Km).

**TABLE 3:** Bacterial strains and plasmids used in this study

Bacterial strain or plasmid	Description^*a*^	Source or reference
Bacterial strains		
S. epidermidis RP62A	Standard strain of S. epidermidis, biofilm positive	ATCC
S. epidermidis 1457	Clinical strain, biofilm positive	21
Δ*vraSR* mutant	*vraSR* deletion mutant derived from SE1457	This study
Δ*vraSR*(pRAB11-*vraSR*) mutant	Δ*vraSR* mutant complemented with plasmid pRAB11-*vraSR*	This study
Δ*vraSR*(pRAB11) mutant	Δ*vraSR* mutant introduced with plasmid pRAB11	This study
Δ*icaC* mutant	*icaC* gene deletion mutant derived from SE1457	22
Δ*atlE* mutant	*atlE* gene deletion mutant derived from SE1457	27
S. aureus 4220	Restriction negative, modification positive	Gao Fu, University of Hong Kong
E. coli DH5α	*supE44*Δ*lacU169* (80d*lacZ*ΔM15) *hsdR17 recA1 endA1 gyrA96 thi-1 relA1*	Invitrogen
E. coli BL21(DE3)	F^−^ *ompT hsdS*_B_ (r_B_^−^ m_B_^−^) *gal dcm* (DE3)	Invitrogen
		
Plasmids		
pET28a	E. coli expression plasmid; Km^r^	Novagen
pET28a-*vraR*	pET28a harboring the *vraR* gene, used for VraR expression	This study
pKOR1	Temp-sensitive E. coli (Amp^r^)-Staphylococcus (Cm^r^) shuttle vector	Li Ming, Fudan University
pKOR1-Δ*vraSR*	Recombinant plasmid	This study
pRAB11	Shuttle vector; Amp^r^ Cm^r^	41
pRAB11-*vraSR*	The *vraSR* genes were cloned into pRAB11	This study

a Km^r^, kanamycin resistance; Amp^r^, ampicillin resistance; Cm^r^, chloramphenicol resistance.

### Extraction of bacterial DNA.

Genomic DNA of S. epidermidis was extracted as described by Flamm et al., with minor modifications (39). In brief, staphylococcal cells were treated with lysostaphin (20 μg/ml; Sigma Co., St. Louis, MO, USA) and proteinase K (100 μg/ml; Merck KGaA, Darmstadt, Germany) and extracted with phenol and chloroform, and the nucleic acids were precipitated with ethanol.

Plasmid DNA from E. coli was extracted with a plasmid purification kit (Qiagen, Hilden, Germany) in accordance with the manufacturer’s instructions. After harvesting and resuspension, bacterial cells were lysed under alkaline conditions. The lysate was neutralized by the addition of potassium acetate. The cleared lysate was loaded onto a Qiagen-tip by gravity flow, and then the eluted plasmid DNA was concentrated by isopropanol precipitation. Plasmid DNA from S. epidermidis or S. aureus 4220 was extracted using the same method except for an additional step of lysostaphin treatment.

### Construction of S. epidermidis *vraSR* deletion mutant and complementary strains.

The *vraSR* deletion mutant of SE1457 was constructed by homologous recombination using the temperature-sensitive plasmid pKOR1 as described by Bae and Schneewind, with minor modifications (40). In brief, the 972-bp downstream fragment of *vraSR* was PCR amplified from SE1457 genomic DNA using primer pair *vra*-DS-F/*vra*-DS-R, and the 920-bp upstream fragment of *vraSR* was amplified using primer pair *vra*-US-F/*vra*-US-R (sequences listed in Table 4). PCR products were ligated after digestion with EcoRI and then cloned into pKOR1 vector with BP Clonase enzyme (Invitrogen) to yield replacement plasmid pKOR1-Δ*vraSR*. The recombinant plasmid pKOR1-Δ*vraSR* was successively transferred into E. coli DH5α, S. aureus RN4220, and then SE1457. The allelic replacement was performed as described previously. The *vraSR* deletion mutant (Δ*vraSR*) was verified by PCR, RT-PCR, and sequencing.

**TABLE 4:** Primers used in this study

Method and primer^*a*^	Sequence (5′–3′)	Size of PCR product (bp)	Note^*b*^
Construction of the *vraSR* deletion mutant			
*vra*-DS-F	GGGGACAAGTTTGTACAAAAAAGCAGGATTATTAGCACTTTTCCCAATG	972	attB1
*vra*-DS-R	CCGGAATTCTAATTCAATAAATTATTAAAGGCG		EcoRI
*vra*-US-S	CCGGAATTCTAGTGATTCATCTGTAAATC	920	EcoRI
*vra*-US-R	GGGGACCACTTTGTACAAGAAAGCTGGGTTAGATTTAGTAAACAATCAA		attB2

Verification of the *vraSR* deletion mutant			
vra-con-F	GGGTCCTTTGCATTCGGTTAC	2,200	
vra-con-R	ACGGCACTGCTTATGTGAACG		

Cotranscription analysis of *vraSR* operon			
V-12F	ATCAGTATTTGAACCAGAAG	519	
V-12R	ATTATTCACCAAAGAAAAGT		
V-23F	ACAAAAGAAAGTACCAATGA	534	
V-23R	TATCGTCCATAAGTAAATCC		
V-34F	TAAGGAAACAAATACCATAG	641	
V-34R	ATATTGTAGTTTTCATTAGTTA		
V-45F	AATCTATTGTGCTGAGGAAA	435	
V-45R	AAATAGAATAATAATCGTGT		

*vraSR* complementation			
pRAB11-*vra*-F	CGGGGTACCAAGATTGAGAATAACCATG		KpnI
pRAB11-*vra*-R	CCGGAATTCTTATTGAATTAAATTATGCTGG		EcoRI

VraR expression			
pET28a-*vraR*-F	CGGGATCCGTGGCGATTAAAGTTTTATTTG	630	BamHI
pET28a-*vraR*-R	CCGCTCGAGTTGAATTAAATTATGCTGGAAC		XhoI

Amplification of promoter fragments			
P-vra-F	TGAAACTAAAGATAAAAG	281	
P-vra-R	TTGTTGTAAATATAAATCT		
P-ica-F	ATTCTAAAATCTCCCCCT		
P-ica1-R	CTTGTTGATGTTCAGATT	301	
P-ica2-R	GGTAGGTGAAAAAATGCA	170	
P-ica3-R	GTTGTTATACTGAAACAGT	92	
P-ica4-R	GTAATTTTAACTTAATTTTTC	66	
P-pbp2-F	CAAGTTTGTTCCTATTTT	329	
P-pbp2-R	TTAGATGCCTCCTACTTA		
P-sgtB-F	ATCTGCACTCATTATTTT	302	
P-sgtB-R	ATGGGTTTTCTCCTTTT		
P-murAA-F	CATTACAAGTTCAAGTT	252	
P-murAA-R	TCCACCATTTATTACTA		
P-rpsJ-F	AAGATTCTCGTGAACAATTC	119	
P-rpsJ-R	GATGTCTACACCTGATGG		

a The primers were designed according to the genomic sequence of S. epidermidis RP62A (GenBank accession number NC_002976). F, forward primer; R, reverse primer.

b Underlined sequences represent the BP Clonase reaction sites or restriction enzyme sites.

For complementation of the Δ*vraSR* mutant, the *vraSR* gene with the associated Shine-Dalgarno sequence in SE1457 was amplified by PCR with primers pRAB11-*vra*-F/pRAB11-*vra*-R. pRAB11-*vraSR* was constructed from pRAB11 inserted with a fragment of *vraSR* digested with KpnI and EcoRI (41). The complementary plasmid was transferred into the Δ*vraSR* mutant by electroporation, yielding the Δ*vraSR*(pRAB11-*vraSR*) complementary strain. The vector plasmid pRAB11 was introduced as a blank control into the Δ*vraSR* mutant and SE1457 and named the Δ*vraSR*(pRAB11) and SE1457(pRAB11) strain, respectively. The *vraSR* complemented strain harboring vector pRAB11 was grown in anhydrotetracycline (50 ng/ml) to induce *vraSR* expression.

### Antimicrobial susceptibility testing.

The susceptibility of SE1457 isogenic *vraSR* mutant strains to antibiotics was determined using the broth dilution method and the Kirby-Bauer disk diffusion test according to Clinical and Laboratory Standards Institute (CLSI) guidelines (17, 42). In brief, S. epidermidis strains were subcultured at least twice, and then a log-phase bacterial culture (6 h) was collected. The turbidity of the bacterial suspension was adjusted to an 0.5 McFarland standard (1.5 × 10^8^ CFU/ml). For the broth dilution assay, the bacterial suspension was inoculated at 1:200 into 2 ml Mueller-Hinton (MH) broth (Oxoid, Basingstoke, UK) containing 2-fold serial dilutions of antibiotics and incubated at 37°C for 16 to 20 h. The MIC was recorded as the lowest concentration of drug in the tube (15 by 18 cm) with complete inhibition of growth by the naked eye. The broth with no drugs served as a blank control. For the disk diffusion assay, the bacterial suspension was spread onto the prepared MH agar (20 ml/petri dish) using a sterile cotton swab, followed by application of paper disks impregnated with appropriate antibiotics after air drying at room temperature for 5 to 10 min. The diameter of the zone of inhibition around each disk was measured after 24 h of incubation at 37°C. Both tests were performed in triplicate for SE1457 isogenic *vraSR* mutant strains.

To detect the susceptibility of the Δ*vraSR* mutant to H_2_O_2_ and SDS stress, overnight cultures of S. epidermidis strains were diluted (1:200) in fresh TSB medium and grown to logarithmic phase (6 h) at 37°C. Bacterial cultures (OD_600,_ 2.5) were serially diluted (1:10), and an aliquot (5 μl) was spotted onto a TSA plate containing 6 mM H_2_O_2_ or 0.2 mM SDS and then incubated at 37°C for 24 h. The bacterial colonies on the TSA plate were photographed.

### Growth curves.

The growth curves of S. epidermidis strains were determined by measuring the OD_600_. Overnight cultures were diluted (1:200) into 20 ml TSB medium with (1, 2, or 4 μg/ml) or without vancomycin and incubated at 37°C with shaking at 220 rpm. The OD_600_ values of the cultures were measured at 60-min intervals for 12 h.

### Microtiter plate assay of *in vitro* biofilm formation.

The biofilm-forming ability of S. epidermidis strains *in vitro* was determined by a semiquantitative plate assay (43). In brief, overnight cultures of SE1457, Δ*vraSR*, Δ*vraSR*(pRAB11-*vraSR*), SE1457(pRAB11), and Δ*vraSR*(pRAB11) strains were diluted with TSB medium containing 0.5% glucose. Aliquots (200 μl per well) were inoculated into a polystyrene 96-well microplate (Corning, Inc., NY, USA) and incubated statically at 37°C for 8, 16, 24, and 48 h. After incubation, the plates were gently washed with phosphate-buffered saline (PBS), fixed with methanol for 15 min, and stained with 2% crystal violet for 5 min. The optical density at 570 nm was measured using a spectrophotometer (Synergy HT; Bio-Tek, USA). Three independent experiments were carried out.

### Assay of *in vivo* biofilm formation.

The biofilm-forming ability of S. epidermidis strains *in vivo* was determined by using a New Zealand rabbit subcutaneous foreign body infection model as described by Wu et al. (21, 44). In brief, disks were cut from polyethylene 8-well strips (8-mm diameter, 1-mm thickness, with a 2-mm projecting rim or chimb) and sterilized by 75% ethanol and UV light. The rabbit (2.0 to 2.5 kg, female) was anesthetized with pentobarbital sodium (5 mg/kg intravenously), and four incisions (10 mm) were made on the back bilaterally along the spine; the subcutis was then carefully dissected to form a cavity (2 cm by 3 cm). In each cavity, three disks were implanted and 1 ml of bacteria (about 10^8^ CFU) suspended in fresh TSB was injected. The same volume of TSB was injected as a control.

Seventy-two hours after staphylococcal inoculation, the rabbits were euthanized and the implants were taken out, gently rinsed with PBS, and observed under a scanning electron microscope (SEM). The biofilms were scraped from the disks, and the viable bacteria were determined by CFU counting as previously described. Three independent experiments were carried out.

### Biofilms observed by CLSM and SEM.

For observation of bacterial biofilms by confocal laser scanning microscopy (CLSM) (model TCS SP5; Leica, Mannheim, Germany), overnight cultures of the SE1457, Δ*vraSR*, and Δ*vraSR*(pRAB11-*vraSR*) strains were inoculated into 2 ml TSB (containing 0.5% glucose) in Fluorodishes (FD35-100; WPI, Sarasota, FL, USA) and incubated statically at 37°C for 24 h (45, 46). The biofilms on the dishes were then rinsed gently with PBS, stained with SYTO9 and propidium iodide (PI) (Live/Dead kit; Invitrogen, Carlsbad, CA, USA), and then observed under a CLSM. The Z-stack composite confocal photomicrographs of viable cells (green) and dead cells (red) were generated using Leica LAS AF software. The fluorescence of live and dead bacteria was quantified using ImageJ software. At least three independent experiments were carried out.

For observation of bacterial biofilms by scanning electron microscopy (SEM) (JSM-6700F; JEOL, Tokyo, Japan), each of the S. epidermidis strains was cultured in a 6-well plate (35-mm diameter, 3 disks/well). After 24 h of incubation at 37°C, the disks were rinsed with PBS, fixed with 2.5% glutaraldehyde in PBS, vacuum dried for 72 h, sputtered with platinum, and then observed under a field emission source instrument.

### Morphology of the Δ*vraSR* mutant observed by TEM.

The SE1457, Δ*vraSR*, and Δ*vraSR*(pRAB11-*vraSR*) strains were cultured in TSB at 37°C for 6 h. The bacterial cells were rinsed with phosphate-buffered saline (PBS) and fixed with 2.5% glutaraldehyde at 4°C for 2 h, followed by 1% osmium for 3 h. The samples were dehydrated in a graded ethanol series, embedded in epoxy resin, and stained using uranyl acetate and lead citrate. Ultrathin sections were cut and then observed under a transmission electron microscope (TEM) (Philips Tecnai-12 Biotwin) (47). The thickness of the cell wall was analyzed using Image-Pro Plus 6.0 software (Media Cybernetics, Inc., Rockville, MD, USA). The thickness of the cell wall was measured at the same 10 positions of each cell, eight cells were measured for each strain, and the thickness of the cell wall was then expressed as the mean value (in nanometers) ± SD.

### Assay of initial adherence capacity.

The primary attachment of SE1457 isogenic *vraSR* mutant strains to a polystyrene surface was determined by crystal violet staining with a modification (35, 47). Mouse serum (1:100) and BSA (10 μg/μl) were diluted with PBS and pipetted into a 96-well microplate, which was then coated at 4°C overnight, with PBS designated as the control. SE1457, Δ*vraSR*, Δ*vraSR*(pRAB11-*vraSR*), and Δ*vraSR*(pRAB11) staphylococcal strains grown at 37°C to an OD_600_ of 1.0 were pipetted into the coated microplate (200 μl/well) and incubated at 37°C for 2 h, followed by washing with PBS, and the subsequent procedures were the same as those used for the semiquantitative biofilm formation assay measuring OD_570_ using a spectrophotometer. Three independent experiments were carried out.

### Assay of PIA in biofilms.

Polysaccharide intercellular adhesin (PIA) in the biofilms of SE1457 isogenic *vraSR* mutant strains was semiquantified by dot blot assay with wheat germ agglutinin (WGA-horseradish peroxidase [HRP] conjugate) as described by Gerke et al. (21, 47, 48). In brief, S. epidermidis strains were subinoculated (1:200) into a 6-well plate (Nunc, Roskilde, Denmark) and incubated at 37°C for 24 h. Biofilms were collected from the bottom of the wells, resuspended in 0.5 M EDTA (3 μl/mg wet weight), and centrifuged (13,000 × *g*, 5 min) after heating at 100°C for 5 min. The supernatant was treated with proteinase K (20 mg/ml) at 37°C for 3 h and inactivated at 100°C for 5 min. Serial dilutions of the PIA extract were transferred to a nitrocellulose membrane (Millipore, Billerica, MA, USA) using a 96-well dot blot device (Biometra GmbH, Göttingen, Germany). The membrane was air dried, blocked with 5% (wt/vol) skim milk, and then incubated with WGA (3.2 μg/ml) conjugated for 1 h with HRP (WGA-HRP conjugate; Lectinotest Laboratory, Lviv, Ukraine). HRP activity was visualized via chromogenic detection using 4-chloride-1-naphthol (Sigma, St. Louis, MO, USA) as the substrate. The quantitation (titer) of PIA was represented as the highest dilution of the supernatant in which HRP was detectable.

### Quantification of eDNA.

The isolation of extracellular DNA (eDNA) from biofilms was performed as described previously (45, 49). In brief, the 24-h biofilms cultured in a 96-well polystyrene plate were chilled at 4°C for 1 h, and EDTA was added at a final concentration of 2.5 mM. After measurement of the OD_600_ of the unwashed biofilm (biofilm biomass), eDNA extraction solution (50 mM Tris-HCl, 10 mM ETDA, 500 mM NaCl, pH 8.0) was added to the wells. The biofilms were scraped off and centrifuged (13,000 × *g*) for 5 min at 4°C. The eDNA in the supernatant was extracted with phenol-chloroform-isoamyl alcohol (25:24:1), precipitated with 100% alcohol, and resuspended in TE buffer.

The amount of eDNA was quantified by qPCR with SYBR Premix Ex Taq (TaKaRa Bio, Inc., Shiga, Japan), using *gyrB* (gyrase B), *serp0306* (ferrichrome transport ATP-binding protein A), *leuA* (2-isopropylmalate synthase), and *lysA* (diaminopimelate decarboxylase A) primers as listed in Table 4. Each gene in the qPCR was assayed in triplicate for three independent experiments. The relative quantitation of eDNA in each sample was calculated as the total eDNA (ng) divided by the biofilm biomass (OD_600_).

### RNA isolation and RNA sequencing.

Total RNA was isolated from SE1457 and Δ*vraSR* strains using an RNeasy minikit (Qiagen, Hilden, Germany) according to the manufacturer’s instructions. In brief, bacterial cells were harvested after 6 h of incubation at 37°C with shaking. The cell pellets were washed with ice-cold 0.85% NaCl and then homogenized 5 times using 0.1-mm zirconia-silica beads in a Mini-Beadbeater (Biospec, Bartlesville, OK, USA) at a speed of 4,800 rpm for 40 s at 1-min intervals on ice. The RNA eluted from the silica-based filter was extracted with phenol-chloroform-isoamyl alcohol and then precipitated with absolute ethanol.

RNA-Seq analysis was performed according to the Illumina RNA sequencing sample preparation guide with three biological replicates for each strain as previously described by Wang et al. (47). To prevent contamination with genomic DNA, samples of SE1457 and Δ*vraSR* mutant RNA were digested with RNase-free DNase I (Sigma, St. Louis, MO, USA). The RNA quality was assessed using a Bioanalyzer system (Agilent Technologies Deutschland GmbH). After removal of rRNA, RNA was fragmented and PCR amplified using random primers. The cDNA libraries were prepared by using an mRNA-Seq sample preparation kit (Illumina, USA). Purified cDNA libraries were quantified using a Qubit fluorometer (Life Technologies, USA), the fragments of 200 to 300 bp were validated using a Bioanalyzer 2100 system (Agilent Technologies, USA), and then sequencing was conducted with an Illumina HiSeq 2500 sequencer for 51 cycles according to the manufacturer’s protocols. Raw sequencing data were analyzed using data collection software provided by Illumina.

### qRT-PCR.

The RNA extracted from SE1457 and the Δ*vraSR* mutant was treated with DNase I and reverse transcribed into cDNA using iScript reverse transcriptase (Bio-Rad, Hercules, CA, USA) by incubation for 5 min at 25°C, followed by 30 min at 42°C and 5 min at 85°C. Then, quantitative PCRs (qPCRs) were performed using SYBR green PCR reagents (Premix Ex Taq; TaKaRa Biotechnology, Dalian, China) in a Mastercycler realplex system (Eppendorf AG, Hamburg, Germany). The amplification conditions were 95°C for 30 s, 40 cycles of 95°C for 5 s, and 60°C for 34 s, followed by melting curve analysis. A *gyrB* (DNA gyrase subunit B) housekeeping gene was used as an internal control. All quantitative real-time reverse transcription-PCRs (qRT-PCRs) were performed in triplicate with at least three independent RNA samples. The sequences of the primers were designed using Beacon Designer software (Premier Biosoft International Ltd., Palo Alto, CA, USA) and are listed in Table 4.

### Expression and purification of recombinant VraR.

A recombinant expression plasmid (pET28a-*vraR*) was constructed by inserting the *vraR* fragment amplified from SE1457 with the primers pET28a-*vraR*-F/pET28a-*vraR*-R (listed in Table 4) into the vector pET28a (+). The plasmid pET28a-*vraR* was transformed into E. coli BL21(DE3). When the transformant was grown to an OD_600_ value of 0.6 at 37°C, 0.8 mM isopropyl β-d-1-thiogalactopyranoside (IPTG) was added for overnight incubation at 22°C. The cells resuspended in lysis buffer (50 mM NaH_2_PO_4_, pH 8.0, 300 mM NaCl, 0.1 mM EDTA, 1 mM phenylmethylsulfonyl fluoride [PMSF]) were sonicated and centrifuged at 15,000 × *g* for 30 min, and the supernatants were loaded onto a nickel-nitrilotriacetic acid column (Qiagen GmbH, Hilden, Germany). His-tagged VraR (6×His-VraR) was eluted using a linear gradient of 30 to 300 mM imidazole, and the protein concentration was determined using a Bradford protein quantification kit (Tiangen, Beijing, China).

### Protein-DNA interactions.

To determine the interaction between VraR and the promoter regions of putative target genes, an electrophoresis mobility shift assay (EMSA) was carried out using a digoxigenin gel shift kit (Roche Diagnostics GmbH, Mannheim, Germany) according to the manufacturer’s instructions. In brief, the predicted promoter regions of *vraSR*, *ica*, *pbp2*, *sgtB*, and *murAA* (66- to 329-bp fragments) were amplified by PCR with the primers listed in Table 4. The DNA fragments were purified using a gel extraction kit (Qiagen, Hilton, Germany) and labeled with digoxigenin using terminal transferase. Purified His-tagged VraR was phosphorylated (VraR-P) by incubation with 50 mM acetylphosphate (Sigma, St. Louis, MO, USA) for 1 h at room temperature. Each gel shift assay included the probe labeled with digoxigenin plus increasing concentrations of VraR-P (ranging from 1.2 to 0.3 μM in a 2-fold dilution), a 125-fold molar excess of the unlabeled specific probe as a competitor was added into the labeled probe plus 1.2 μM VraR-P, and a 125-fold molar excess of unlabeled nonspecific DNA (119-bp coding sequence of *rpsJ*) as a negative control was added into the labeled probe plus 1.2 μM VraR-P. All samples were incubated at 25°C for 20 min, separated by electrophoresis on a 6% nondenaturing polyacrylamide gel, and blotted onto a positively charged nylon membrane (Millipore, Bedford, MA, USA). The blots were incubated with alkaline phosphatase-conjugated anti-digoxigenin antibody, followed by chloro-5-substituted adamantyl-1,2-dioxetane phosphate (CSPD) solution for chemiluminescent detection, and exposed to X-ray film.

### Ethics statement.

All animal experiment procedures were carried out according to relevant national and international guidelines (the Regulations for the Administration of Affairs Concerning Experimental Animals, China, and the NIH Guide for the Care and Use of Laboratory Animals) and were approved by the Institutional Animal Care and Use Committee (IACUC) of the School of Basic Medicine, Dali University (no. 201809280311).

### Statistical analysis.

Data from the susceptibility assay, biofilm assay, initial attachment assay, and eDNA assay were analyzed by GraphPad Prism software (San Diego, CA, USA) using the Student's *t* test. Differences with a *P* value of less than 0.05 were considered statistically significant.

## References

[1] O'Connor AM, McManus BA, Kinnevey PM, Brennan GI, Fleming TE, Cashin PJ, O'Sullivan M, Polyzois I, Coleman DC. 2018. Significant enrichment and diversity of the staphylococcal arginine catabolic mobile element ACME in *Staphylococcus epidermidis* isolates from subgingival peri-implantitis sites and periodontal pockets. Front Microbiol 9:1558. doi:10.3389/fmicb.2018.01558. 30050526 PMC6052350

[2] Igbinosa EO, Beshiru A, Akporehe LU, Ogofure AG. 2016. Detection of methicillin-resistant staphylococci isolated from food producing animals: a public health implication. Vet Sci 3:14. doi:10.3390/vetsci3030014. PMC5606580 29056723

[3] Fey PD, Olson ME. 2010. Current concepts in biofilm formation of Staphylococcus epidermidis. Future Microbiol 5:917–933. doi:10.2217/fmb.10.56. 20521936 PMC2903046

[4] Otto M. 2017. Staphylococcus epidermidis: a major player in bacterial sepsis. Future Microbiol 12:1031–1033. doi:10.2217/fmb-2017-0143. 28748707 PMC5627029

[5] Mann EE, Rice KC, Boles BR, Endres JL, Ranjit D, Chandramohan L, Tsang LH, Smeltzer MS, Horswill AR, Bayles KW. 2009. Modulation of eDNA release and degradation affects *Staphylococcus aureus* biofilm maturation. PLoS One 4:e5822. doi:10.1371/journal.pone.0005822. 19513119 PMC2688759

[6] Taglialegna A, Varela MC, Rosato RR, Rosato AE. 2019. VraSR and virulence trait modulation during daptomycin resistance in methicillin-resistant *Staphylococcus aureus* infection. mSphere 4:e00557-18. doi:10.1128/mSphere.00557-18. 30760612 PMC6374592

[7] Gao C, Dai Y, Chang W, Fang C, Wang Z, Ma X. 2019. VraSR has an important role in immune evasion of *Staphylococcus aureus* with low level vancomycin resistance. Microbes Infect 21:361–367. doi:10.1016/j.micinf.2019.04.003. 31009806

[8] Sengupta M, Jain V, Wilkinson BJ, Jayaswal RK. 2012. Chromatin immunoprecipitation identifies genes under direct VraSR regulation in *Staphylococcus aureus*. Can J Microbiol 58:703–708. doi:10.1139/w2012-043. 22571705

[9] Mehta S, Cuirolo AX, Plata KB, Riosa S, Silverman JA, Rubio A, Rosato RR, Rosato AE. 2012. VraSR two-component regulatory system contributes to *mprF*-mediated decreased susceptibility to daptomycin in *in vivo*-selected clinical strains of methicillin-resistant *Staphylococcus aureus*. Antimicrob Agents Chemother 56:92–102. doi:10.1128/AAC.00432-10. 21986832 PMC3256076

[10] Belcheva A, Golemi-Kotra D. 2008. A close-up view of the VraSR two-component system. A mediator of *Staphylococcus aureus* response to cell wall damage. J Biol Chem 283:12354–12364. doi:10.1074/jbc.M710010200. 18326495

[11] Yin S, Daum RS, Boyle-Vavra S. 2006. VraSR two-component regulatory system and its role in induction of *pbp2* and *vraSR* expression by cell wall antimicrobials in *Staphylococcus aureus*. Antimicrob Agents Chemother 50:336–343. doi:10.1128/AAC.50.1.336-343.2006. 16377706 PMC1346790

[12] Boyle-Vavra S, Yin S, Jo DS, Montgomery CP, Daum RS. 2013. VraT/YvqF is required for methicillin resistance and activation of the VraSR regulon in *Staphylococcus aureus*. Antimicrob Agents Chemother 57:83–95. doi:10.1128/AAC.01651-12. 23070169 PMC3535960

[13] Jo DS, Montgomery CP, Yin S, Boyle-Vavra S, Daum RS. 2011. Improved oxacillin treatment outcomes in experimental skin and lung infection by a methicillin-resistant *Staphylococcus aureus* isolate with a *vraSR* operon deletion. Antimicrob Agents Chemother 55:2818–2823. doi:10.1128/AAC.01704-10. 21383093 PMC3101413

[14] Dai Y, Gao C, Chen L, Chang W, Yu W, Ma X, Li J. 2019. Heterogeneous vancomycin-intermediate *Staphylococcus aureus* uses the VraSR regulatory system to modulate autophagy for increased intracellular survival in macrophage-like cell line RAW264.7. Front Microbiol 10:1222. doi:10.3389/fmicb.2019.01222. 31214151 PMC6554704

[15] Gardete S, Wu SW, Gill S, Tomasz A. 2006. Role of VraSR in antibiotic resistance and antibiotic-induced stress response in *Staphylococcus aureus*. Antimicrob Agents Chemother 50:3424–3434. doi:10.1128/AAC.00356-06. 17005825 PMC1610096

[16] McCallum N, Meier PS, Heusser R, Berger-Bachi B. 2011. Mutational analyses of open reading frames within the *vraSR* operon and their roles in the cell wall stress response of *Staphylococcus aureus*. Antimicrob Agents Chemother 55:1391–1402. doi:10.1128/AAC.01213-10. 21220524 PMC3067146

[17] Kato Y, Suzuki T, Ida T, Maebashi K. 2010. Genetic changes associated with glycopeptide resistance in *Staphylococcus aureus*: predominance of amino acid substitutions in YvqF/VraSR. J Antimicrob Chemother 65:37–45. doi:10.1093/jac/dkp394. 19889788 PMC2800785

[18] Otto M. 2013. Staphylococcal infections: mechanisms of biofilm maturation and detachment as critical determinants of pathogenicity. Annu Rev Med 64:175–188. doi:10.1146/annurev-med-042711-140023. 22906361

[19] Gotz F. 2002. Staphylococcus and biofilms. Mol Microbiol 43:1367–1378. doi:10.1046/j.1365-2958.2002.02827.x. 11952892

[20] Otto M. 2008. Staphylococcal biofilms. Curr Top Microbiol Immunol 322:207–228. doi:10.1007/978-3-540-75418-3_10. 18453278 PMC2777538

[21] Wu Y, Wu Y, Zhu T, Han H, Liu H, Xu T, Francois P, Fischer A, Bai L, Götz F, Qu D. 2015. *Staphylococcus epidermidis* SrrAB regulates bacterial growth and biofilm formation differently under oxic and microaerobic conditions. J Bacteriol 197:459–476. doi:10.1128/JB.02231-14. 25404696 PMC4285975

[22] Wu Y, Wang J, Xu T, Liu J, Yu W, Lou Q, Zhu T, He N, Ben H, Hu J, Gotz F, Qu D. 2012. The two-component signal transduction system ArlRS regulates *Staphylococcus epidermidis* biofilm formation in an *ica*-dependent manner. PLoS One 7:e40041. doi:10.1371/journal.pone.0040041. 22848368 PMC3407220

[23] Yamada KJ, Kielian T. 2019. Biofilm-leukocyte cross-talk: impact on immune polarization and immunometabolism. J Innate Immun 11:280–288. doi:10.1159/000492680. 30347401 PMC6476693

[24] Somerville GA, Cockayne A, Durr M, Peschel A, Otto M, Musser JM. 2003. Synthesis and deformylation of *Staphylococcus aureus* delta-toxin are linked to tricarboxylic acid cycle activity. J Bacteriol 185:6686–6694. doi:10.1128/JB.185.22.6686-6694.2003. 14594843 PMC262117

[25] Grosser MR, Weiss A, Shaw LN, Richardson AR. 2016. Regulatory requirements for *Staphylococcus aureus* nitric oxide resistance. J Bacteriol 198:2043–2055. doi:10.1128/JB.00229-16. 27185828 PMC4944221

[26] Kuroda M, Kuwahara-Arai K, Hiramatsu K. 2000. Identification of the up- and down-regulated genes in vancomycin-resistant *Staphylococcus aureus* strains Mu3 and Mu50 by cDNA differential hybridization method. Biochem Biophys Res Commun 269:485–490. doi:10.1006/bbrc.2000.2277. 10708580

[27] Qin Z, Ou Y, Yang L, Zhu Y, Tolker NT, Molin S, Qu D. 2007. Role of autolysin-mediated DNA release in biofilm formation of *Staphylococcus epidermidis*. Microbiology (Reading) 153:2083–2092. doi:10.1099/mic.0.2007/006031-0. 17600053

[28] Levinger O, Bikels-Goshen T, Landau E, Fichman M, Shapira R. 2012. Epigallocatechin gallate induces upregulation of the two-component VraSR system by evoking a cell wall stress response in *Staphylococcus aureus*. Appl Environ Microbiol 78:7954–7959. doi:10.1128/AEM.02253-12. 22941085 PMC3485954

[29] Kuroda M, Kuroda H, Oshima T, Takeuchi F, Mori H, Hiramatsu K. 2003. Two-component system VraSR positively modulates the regulation of cell-wall biosynthesis pathway in *Staphylococcus aureus*. Mol Microbiol 49:807–821. doi:10.1046/j.1365-2958.2003.03599.x. 12864861

[30] Rice KC, Mann EE, Endres JL, Weiss EC, Cassat JE, Smeltzer MS, Bayles KW. 2007. The CidA murein hydrolase regulator contributes to DNA release and biofilm development in Staphylococcus aureus. Proc Natl Acad Sci USA 104:8113–8118. doi:10.1073/pnas.0610226104. 17452642 PMC1876580

[31] Rice KC, Firek BA, Nelson JB, Yang SJ, Patton TG, Bayles KW. 2003. The *Staphylococcus aureus* cidAB operon: evaluation of its role in regulation of murein hydrolase activity and penicillin tolerance. J Bacteriol 185:2635–2643. doi:10.1128/JB.185.8.2635-2643.2003. 12670989 PMC152627

[32] Mashruwala AA, Guchte AV, Boyd JM. 2017. Impaired respiration elicits SrrAB-dependent programmed cell lysis and biofilm formation in *Staphylococcus aureus*. Elife 6:e23845. doi:10.7554/eLife.23845. 28221135 PMC5380435

[33] Ranjit DK, Endres JL, Bayles KW. 2011. *Staphylococcus aureus* CidA and LrgA proteins exhibit holin-like properties. J Bacteriol 193:2468–2476. doi:10.1128/JB.01545-10. 21421752 PMC3133170

[34] Bayles KW. 2007. The biological role of death and lysis in biofilm development. Nat Rev Microbiol 5:721–726. doi:10.1038/nrmicro1743. 17694072

[35] Zhu T, Lou Q, Wu Y, Hu J, Yu F, Qu D. 2010. Impact of the *Staphylococcus epidermidis* LytSR two-component regulatory system on murein hydrolase activity, pyruvate utilization and global transcriptional profile. BMC Microbiol 10:287. doi:10.1186/1471-2180-10-287. 21073699 PMC2996381

[36] Windham IH, Chaudhari SS, Bose JL, Thomas VC, Bayles KW. 2016. SrrAB modulates *Staphylococcus aureus* cell death through regulation of *cidABC* transcription. J Bacteriol 198:1114–1122. doi:10.1128/JB.00954-15. 26811317 PMC4800867

[37] Dengler V, Meier PS, Heusser R, Berger-Bachi B, McCallum N. 2011. Induction kinetics of the *Staphylococcus aureus* cell wall stress stimulon in response to different cell wall active antibiotics. BMC Microbiol 11:16. doi:10.1186/1471-2180-11-16. 21251258 PMC3032642

[38] Gill SR, Fouts DE, Archer GL, Mongodin EF, Deboy RT, Ravel J, Paulsen IT, Kolonay JF, Brinkac L, Beanan M, Dodson RJ, Daugherty SC, Madupu R, Angiuoli SV, Durkin AS, Haft DH, Vamathevan J, Khouri H, Utterback T, Lee C, Dimitrov G, Jiang L, Qin H, Weidman J, Tran K, Kang K, Hance IR, Nelson KE, Fraser CM. 2005. Insights on evolution of virulence and resistance from the complete genome analysis of an early methicillin-resistant *Staphylococcus aureus* strain and a biofilm-producing methicillin-resistant *Staphylococcus epidermidis* strain. J Bacteriol 187:2426–2438. doi:10.1128/JB.187.7.2426-2438.2005. 15774886 PMC1065214

[39] Flamm RK, Hinrichs DJ, Thomashow MF. 1984. Introduction of pAM beta 1 into Listeria monocytogenes by conjugation and homology between native L. monocytogenes plasmids. Infect Immun 44:157–161. doi:10.1128/iai.44.1.157-161.1984. 6323313 PMC263486

[40] Bae T, Schneewind O. 2006. Allelic replacement in *Staphylococcus aureus* with inducible counter-selection. Plasmid 55:58–63. doi:10.1016/j.plasmid.2005.05.005. 16051359

[41] Helle L, Kull M, Mayer S, Marincola G, Zelder ME, Goerke C, Wolz C, Bertram R. 2011. Vectors for improved Tet repressor-dependent gradual gene induction or silencing in *Staphylococcus aureus*. Microbiology (Reading) 157:3314–3323. doi:10.1099/mic.0.052548-0. 21921101

[42] Turnidge J, Bordash G. 2007. Statistical methods for establishing quality control ranges for antibacterial agents in Clinical and Laboratory Standards Institute susceptibility testing. Antimicrob Agents Chemother 51:2483–2488. doi:10.1128/AAC.01457-06. 17438045 PMC1913260

[43] Christensen GD, Simpson WA, Younger JJ, Baddour LM, Barrett FF, Melton DM, Beachey EH. 1985. Adherence of coagulase-negative staphylococci to plastic tissue culture plates: a quantitative model for the adherence of staphylococci to medical devices. J Clin Microbiol 22:996–1006. doi:10.1128/jcm.22.6.996-1006.1985. 3905855 PMC271866

[44] He N, Hu J, Liu H, Zhu T, Huang B, Wang X, Wu Y, Wang W, Qu D. 2011. Enhancement of vancomycin activity against biofilms by using ultrasound-targeted microbubble destruction. Antimicrob Agents Chemother 55:5331–5337. doi:10.1128/AAC.00542-11. 21844319 PMC3195034

[45] Hu J, Xu T, Zhu T, Lou Q, Wang X, Wu Y, Huang R, Liu J, Liu H, Yu F, Ding B, Huang Y, Tong W, Qu D. 2011. Monoclonal antibodies against accumulation-associated protein affect EPS biosynthesis and enhance bacterial accumulation of *Staphylococcus epidermidis*. PLoS One 6:e20918. doi:10.1371/journal.pone.0020918. 21687690 PMC3110253

[46] Liu H, Zhao D, Chang J, Yan L, Zhao F, Wu Y, Xu T, Gong T, Chen L, He N, Wu Y, Han S, Qu D. 2014. Efficacy of novel antibacterial compounds targeting histidine kinase YycG protein. Appl Microbiol Biotechnol 98:6003–6013. doi:10.1007/s00253-014-5685-8. 24737057 PMC4057637

[47] Wang X, Han H, Lv Z, Lin Z, Shang Y, Xu T, Wu Y, Zhang Y, Qu D. 2017. PhoU2 but not PhoU1 as an important regulator of biofilm formation and tolerance to multiple stresses by participating in various fundamental metabolic processes in *Staphylococcus epidermidis*. J Bacteriol 199:e00219-17. doi:10.1128/JB.00219-17. 28947672 PMC5686610

[48] Gerke C, Kraft A, Sussmuth R, Schweitzer O, Gotz F. 1998. Characterization of the N-acetylglucosaminyltransferase activity involved in the biosynthesis of the *Staphylococcus epidermidis* polysaccharide intercellular adhesin. J Biol Chem 273:18586–18593. doi:10.1074/jbc.273.29.18586. 9660830

[49] Lou Q, Zhu T, Hu J, Ben H, Yang J, Yu F, Liu J, Wu Y, Fischer A, Francois P, Schrenzel J, Qu D. 2011. Role of the SaeRS two-component regulatory system in *Staphylococcus epidermidis* autolysis and biofilm formation. BMC Microbiol 11:146. doi:10.1186/1471-2180-11-146. 21702925 PMC3224141

