# Supplementary figures and images for: The Vancomycin Resistance-Associated Regulatory System VraSR Modulates Biofilm Formation of Staphylococcus epidermidis in an ica-Dependent Manner
Source: mSphere. 2021 Sep 22;6(5):e00641-21. doi: 10.1128/mSphere.00641-21 (PMC8550092; doi:10.1128/mSphere.00641-21)

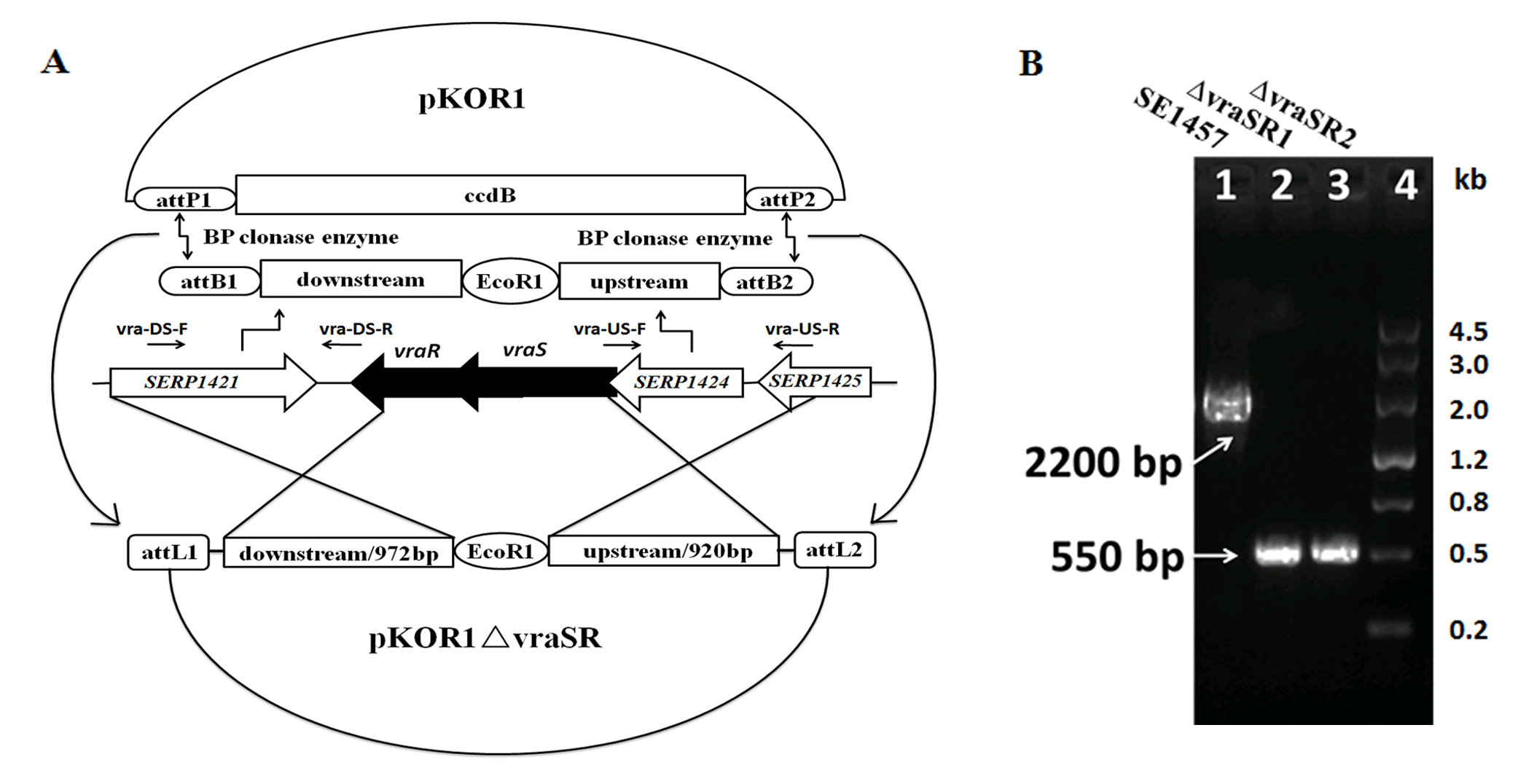

Supplement: FIG S1 [file msphere.00641-21-sf001.tif]

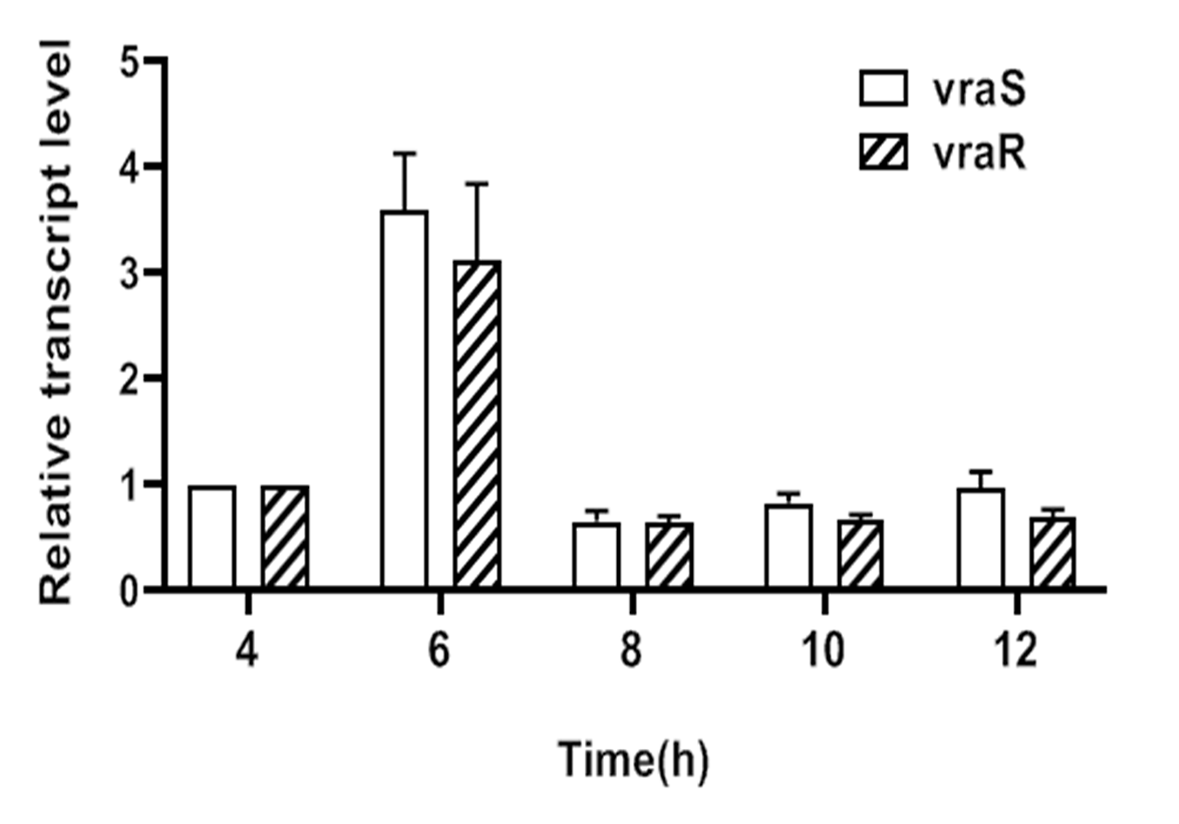

Supplement: FIG S2 [file msphere.00641-21-sf002.tif]

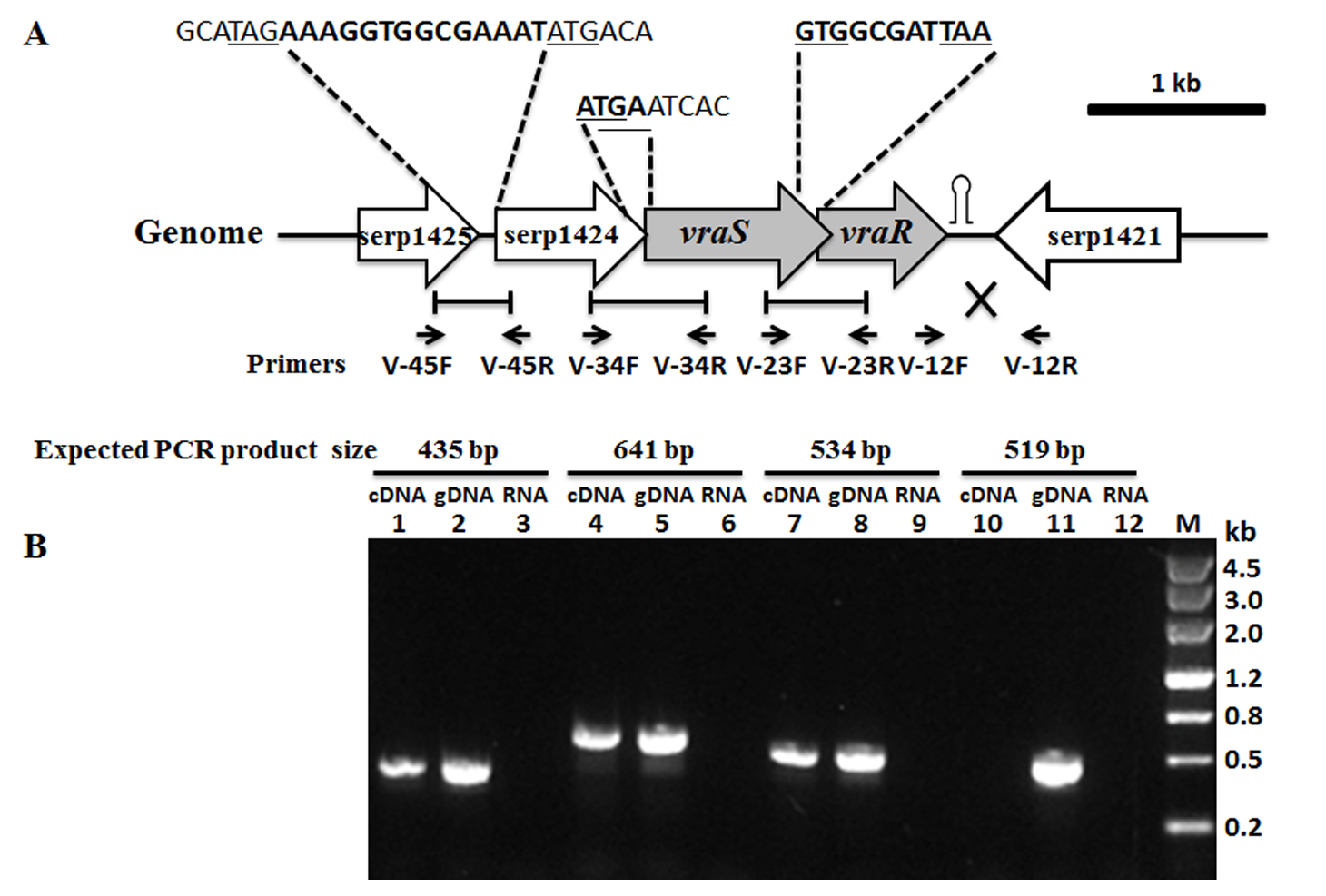

Supplement: FIG S3 [file msphere.00641-21-sf003.tif]

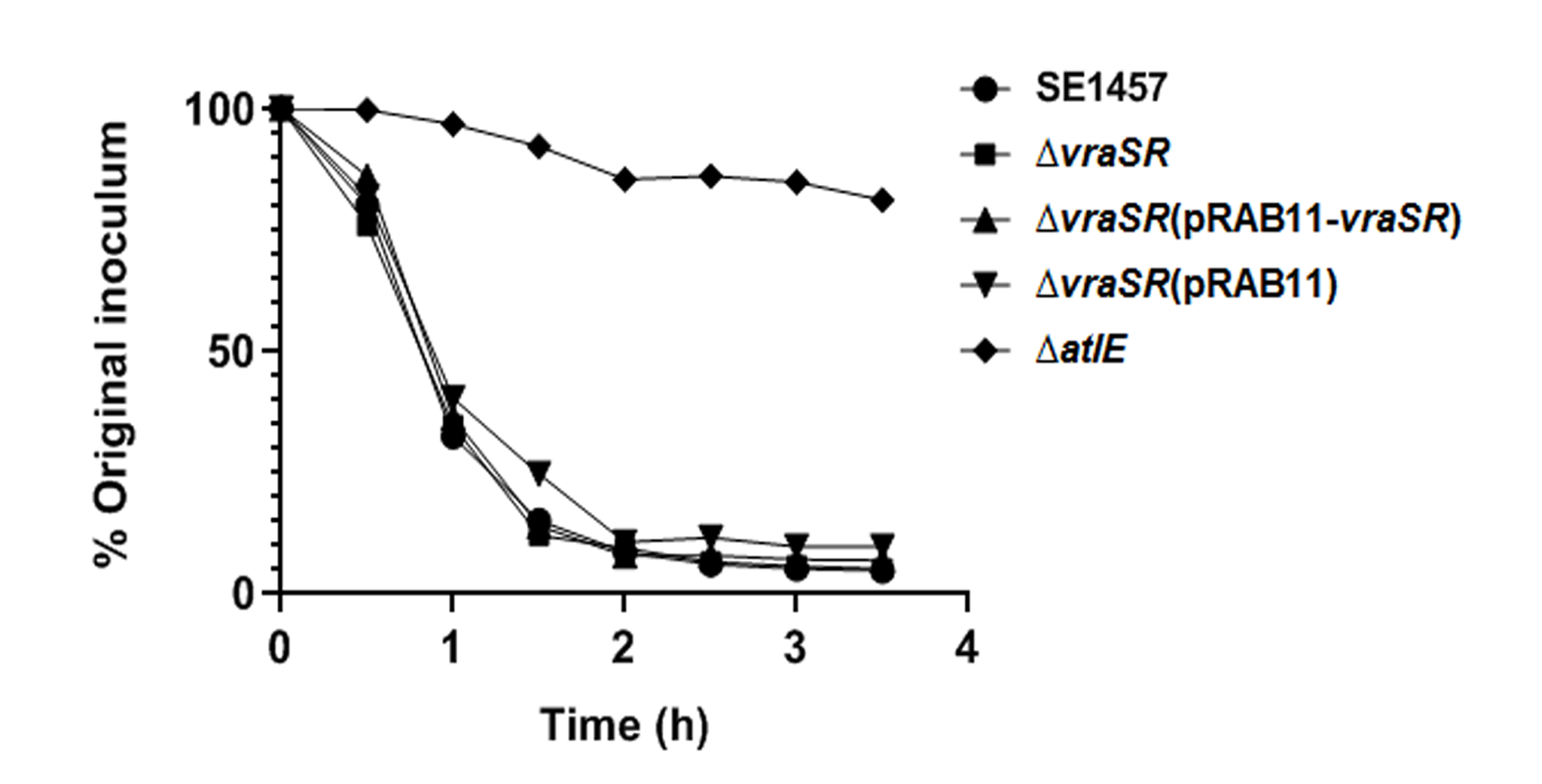

Supplement: FIG S4 [file msphere.00641-21-sf004.tif]
