# Supplementary material for: The Vancomycin Resistance-Associated Regulatory System VraSR Modulates Biofilm Formation of Staphylococcus epidermidis in an ica-Dependent Manner
Source: mSphere. 2021 Sep 22;6(5):e00641-21. doi: 10.1128/mSphere.00641-21 (PMC8550092; doi:10.1128/mSphere.00641-21)
Supplement: TABLE S1 [file msphere.00641-21-st001.docx]

**Supplemental materials**

**Table S1 Antimicrobial susceptibility of the *S. epidermidis* *vraSR* deletion mutant (disk diffusion test)**

| **Antibiotics** | **Inhibition zone (mm)** | | | | |
| --- | --- | --- | --- | --- | --- |
|  | **SE1457** | **∆*vraSR*** | **SE1457**  **(pRAB11)** | **∆*vraSR***  **(pRAB11)** | **∆*vraSR***  **(pRAB11-*vraSR*)** |
| Van | 18.8±0.5 | 21.6±0.94 | 20.5±0.58 | 22.5±1.29 | 21.8±1.31 |
| Am | 45.3±3.6 | 48.5±2.08 | 51±1.73 | 53.5±0.58 | 51±0.58 |
| CXM | 40.25±0.96 | 44.4±1.11 | 41.5±1.29 | 46.3±1.89 | 43.3±0.96 |
| CTX | 35.5±0.58 | 40.5±1.29 | 39±2.16 | 42±2.45 | 41.8±2.22 |
| AK | 26.5±0.58 | 27.4±1.25 | 30.3±1.7 | 29.8±2.75 | 30.8±0.98 |
| GN | 27.5±0.58 | 30.5±5.74 | 30±1.63 | 28.6±1.70 | 31±2.0 |
| TE | 34±1.41 | 35±2.16 | 34.5±3.11 | 36.5±4.43 | 36.5±2.38 |
| LVF | 34.5±1.0 | 34.8±0.98 | 36.3±1.26 | 36.5±1.73 | 37.3±1.89 |

Van: vancomycin; Am: ampicillin; CXM: cefuroxime; CTX: cefotaxime; AK: amikacin; GN: gentamycin; TE: tetracycline; LVF: levofloxacin.
