# Supplementary material for: The Vancomycin Resistance-Associated Regulatory System VraSR Modulates Biofilm Formation of Staphylococcus epidermidis in an ica-Dependent Manner
Source: mSphere. 2021 Sep 22;6(5):e00641-21. doi: 10.1128/mSphere.00641-21 (PMC8550092; doi:10.1128/mSphere.00641-21)
Supplement: TABLE S2 [file msphere.00641-21-st002.docx]

**Supplemental materials**

**Table S2 Analysis of putative promoter regions of genes regulated by VraR**

| **Genes or loci** | **Putative promoter region (5'—3')**  **VraR motif** | **Description** |
| --- | --- | --- |
| *serp1425* | **ACT**AA**AGT** (-85nt ~ -78nt) …**GTG**ATA | Vancomycin-resistance associated regulatory system |
|  | G**TGA**TA**TCA** (+2nt ~ +9nt) TTG … |  |
| *ica* operon | **ACT**GTTTC**AGT**(-92nt ~ -82nt)…**ATG**CAT | Intercellular adhesion locus |
|  | **ATG**CAT…**TGA**ACA**TCA**(+123nt ~ +131nt) |  |
|  | **ATG**CAT…**TGA**AA**TCA**(+273nt ~ +280nt) |  |
|  | **ATG**CAT…**TGA**CT**TCA**(+285nt ~ +292nt) |  |
| *serp1412* | **ACT**CA**AGT**(-92nt ~ -82nt)…**ATG** | Transglycosylase domain protein |
| *murAA* | **ACT**CA**AGT**(-136nt ~ -129nt)…**ATG** | UDP-N-acetylglucosamine-1-carboxyvinyltransferase |
| *lrgA* | **TGA**GTT**TCA**(-124nt ~ -116nt)…**ATG** | antiholin-like murein hydrolase modulator |
| *pflAB* | **ACT**TT**AGT**(-100nt ~ -93nt)…**ATG** | pyruvate formate lyase activating enzyme |
| *serp0331* | **ACT**TA**AGT**(-63nt ~ -57nt)…**ATG** | Hypothetical protein |
| *serp0707* | **ACT**TT**AGT**(-20nt ~ -13nt)…**ATG** | Hypothetical protein |
